# Off-Grid System for Production of Green Hydrogen via Electrolysis of Industrial Effluents: A Technical Analysis

**DOI:** 10.1021/acsomega.5c08182

**Published:** 2026-02-10

**Authors:** Pedro H. L Gomes, João P. M. S Martins, Daniel S. Serra, Tobias P. Tavares, Kelly L. Oliveira, Kelma M. S P Cavalcante, Carla F. Andrade, Carlucio R. Alves, Concepción Caravaca, Rita X. Valenzuela, Mona L. M. Oliveira

**Affiliations:** † Energy Conversion Laboratory, Center of Science and Technology, 67843 State University of Ceará, Campus Itaperi, Fortaleza, CE 60714-903, Brazil; ‡ Department of Mechanical Engineering, 28121 Federal University of Ceará (UFC), Pici Campus, Fortaleza, CE 60455-760, Brazil; § Department of Fisheries Engineering, Federal University of Ceará (UFC), Pici Campus, Fortaleza, CE 60455-760, Brazil; ∥ Functional Materials for Hydrogen Technologies (FUNMATECH2), Centro de Investigaciones Energé'ticas, Medioambientales y Tecnológicas (CIEMAT), Av. Complutense, 40, 28040 Madrid, Spain

## Abstract

This study evaluates, through simulation, the technical feasibility and highlights the environmental benefits of a proposed off-grid system for electrolytic green hydrogen production using industrial effluents and solar photovoltaic and wind energy (Off-Grid GH_2_PS). The simulations compare the operational dynamics of the current scenario (grid electricity and natural gas) of an industry located in northeastern Brazil with those of the proposed system (renewable electricity, PEM electrolyzer, effluent conditioning system, battery storage, and hydrogen–natural gas blending). The results show strong solar–wind complementarity, with irradiance ranging from 4.77–6.92 kWh/m^2^/day and wind speeds from 5.04–8.49 m/s, resulting in a total generation of 1.91 GWh/year, of which 62% came from solar energy. Of this amount, the electrolyzer consumed 1.36 GWh/year (84% of the demand). Hydrogen production reached 17,976 kg/year, and annual consumption totaled 17,262 kg. The 10% hydrogen blending into natural gas reduced natural gas use by 3%, but it required a 6.5% increase in volumetric flow. Effluent conditioning showed low seasonal variability and delivered 486.8 m^3^/year, although only 27% reached the electrolyzer due to purification losses. It was observed that 27.54 kgH_2_O/kgH_2_ and an energy consumption of 75.90 kWh/kgH_2_ were required. Environmental performance showed an emission reduction of 10.99% (465.5 tCO_2_eq/year). Overall, the Off-Grid GH_2_PS demonstrates a strong potential for industrial decarbonization in regions with high availability of renewable resources and effluents. Finally, future studies should incorporate high-resolution temporal data sets, integrate degradation models for system components, and enable dynamic coupling between water treatment and the energy–hydrogen subsystems. A detailed life-cycle assessment is also recommended to strengthen the overall sustainability analysis, as well as an economic feasibility evaluation of the proposed system.

## Graphical abstract

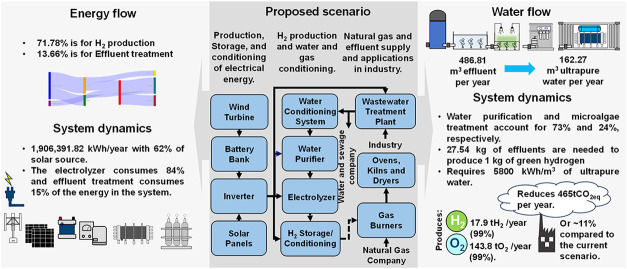

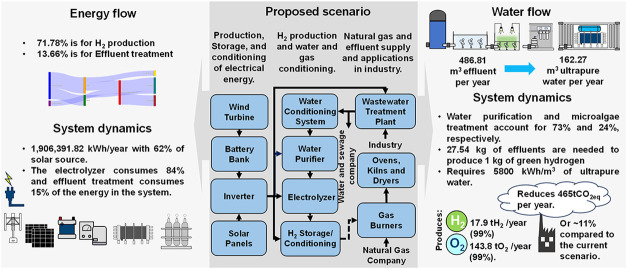

## Introduction

The world is experiencing the consequences of global warming, which has been intensified by anthropogenic pollution over time. In light of this issue, an energy transition has become essential and has led to international treaties such as the Paris Agreement.^1^ In this context, various solutions have emerged to decarbonize different sectors of society, including replacing fossil-based H_2_ production with green hydrogen (GH_2_) via water electrolysis.^2^

Since the past decade, publications in the field of green hydrogen have grown exponentially up to the present day, clearly demonstrating the scientific and technological evolution and challenges of the field, both in terms of technological maturity and system integration, thus positioning green hydrogen as an essential vector for global decarbonization.

In 2012, Dincer^3^ presented one of the pioneering works on green hydrogen production methods, systematizing electrical, thermal, biochemical, and photochemical routes and discussing their environmental and energy potential. A few years later, Nikolaidis and Poullikkas^4^ consolidated one of the most influential comparative reviews, evaluating technical and economic aspects of 14 production processes and highlighting the growing role of pyrolysis and gasification compared to conventional routes.

With advances in electrochemical technologies, Kumar and Himabindu^5^ shifted the focus to PEM electrolysis, detailing catalysts, economic challenges, and the advancements required for commercial feasibility. Around the same period, Kim et al.^6^ expanded the discussion to systems based on photoelectrochemical and photocatalytic routes, proposing the “leaf-to-farm challenge” concept for scaling such systems.

More recently, Ishaq, Dincer, and Crawford^7^ presented an integrated view of the energy transition, addressing blue and green production methods, storage, transport, and the environmental impacts of hydrogen, consolidating the contemporary state of the art.

In this context, emerging technologies such as thermally assisted hybrid electrolysis (SOEC), photoelectrochemical (PEC) processes, and bioelectrochemical approaches have been widely studied as alternatives to reduce the energy consumption of hydrogen production by combining different forms of energy input into the electrochemical process.^8–10^ These approaches may integrate heat, direct sunlight on photoelectrodes, or even the biological oxidation of organic matter, sometimes concurrently with wastewater treatment.^11^ Although these options represent important advances in the state of the art, the present study focuses on a conventional PEM electrolyzer powered by renewable sources (solar + wind) without integrating hybrid mechanisms at the electrochemical cell level.

There are different technologies used for hydrogen production via water electrolysis, including proton exchange membrane electrolyzer (PEMEL), solid oxide electrolysis cell (SOEC), anion exchange membrane electrolyzer (AEMEL), and alkaline electrolyzer (AEL).^12^ In addition to these technologies, some studies have investigated microbial electrolysis cells (MECs) as an alternative route for the production of hydrogen from wastewater. MECs use electroactive microorganisms to oxidize organic matter and drive hydrogen evolution with a small external voltage, enabling simultaneous wastewater treatment and biohydrogen generation.^11^ These technologies produce GH_2_, which is used in applications currently associated with fossil-based hydrogen, such as electricity generation via fuel cells and heat generation in industrial burners.^13,14^

In Brazil, renewable energy sources account for 44.8% of the total energy mix, compared to 15% globally.^15,16^ The Northeast region stands out, with a record median wind energy production of 14,722 MW and a capacity factor of 75.07%.^17^ In Ceará, 81.48% of the state’s electricity generation comes from renewable sources, with wind energy representing 49.98% and solar energy representing 6.24%.^18^ These indicators strengthen and encourage the adoption of these resources for GH_2_ production.

The electrolysis process not only uses energy resources but also requires water, a resource that is often limited. Despite its diverse sources, water is primarily allocated for human and animal consumption.^19^ Therefore, selecting an appropriate water source for hydrogen production is crucial because its origin can increase process costs and potentially make operations unfeasible in certain regions. In this context, industrial effluents emerge as an alternative that does not compete directly with other demands for urban water resources.^20^ Using wastewater as a feedstock for hydrogen production can simultaneously address the energy crisis and water scarcity.^21^

Recent studies demonstrate the feasibility of coupling photovoltaic or hybrid renewable generation with PEM, AEM, or SOEC electrolysis units supplied by treated industrial effluents or municipal wastewater.^12,19–22^ Fakourian and Alizadeh^22^ investigated the amount of electrolyzed water and H_2_ produced under solar irradiation conditions in Texas, and predicted that approximately 1.43·10^4^ Nm^3^/year of hydrogen would come from coal boiler wastewater through an integrated photovoltaic electrolysis system. Barghash et al.,^12^ for example, achieved 50 tH_2_/day using residential effluents and solar electricity with PEMEL.

Other papers highlight the water–energy nexus and the potential of wastewater-based hydrogen production systems,^23–25^ including pilot units that integrate PV-electrolysis using industrial effluents.^26–28^ Cvetković et al.^23^ produced 520 tH_2_/t of effluent during corn starch production with biogas cogeneration in the industrial sector. Additionally, Campana et al.^24^ achieved approximately 36 tH_2_/year using a hybrid wind–solar system at a treatment plant. Jolaoso et al.^25^ produced 242 tH_2_/day from flue gas desulfurization effluents using solar-powered electrolysis.

Various international projects have used effluents to produce GH_2_. Notable European initiatives include a 91 kgH_2_/day plant in Cambridgeshire, England. This plant was developed through a partnership between Anglian Water, Element 2, and Logan Energy.^29,30^ Similarly, the Hydrogen Collective and the Queensland University of Technology project in Goondiwindi, Australia, boasts a capacity of 1350 tH_2_/year.^31^ In Spain, the Andalusian GH_2_ Valley, with Arcgisa facilities, will produce 4.2 million m^3^/year using solar and wind energy. The Arroyo Culebro Cuenca Media Alta plant will generate 80 tH_2_/year using photovoltaic and biogas energy.^32,33^ The partnership between Northern Ireland Water and Lagan MEICA Ltd. in Belfast, Northern Ireland, produces 451 kg H_2_/day.^34^

In North America, the Mendota plant in California produces 30 tH_2_/day using 120 MW of PEMEL and 300 MW of solar energy.^35^ In South America, Ecopetrol in Cartagena, Colombia, generates 20 kg H_2_/day using 50 kW of photovoltaic energy.^36^ In Brazil, the HYDROS project, led by the Ceará Water and Sewage Company (CAGECE) and Utilitas Pecém, supplies effluents to companies at the Pecém GH_2_ Hub.^37^ The reuse water supply will be approximately 4.5 m^3^/s and will meet the demand of the EDP Group plant, which produces 250 N m^3^ H_2_/hour via PEMEL powered by 3 MW of photovoltaic panels.^38^

Scientific research and green hydrogen projects under development worldwide, as described in the previous paragraphs, highlight the growing relevance of the field, including the production of green hydrogen via electrolysis of effluents, for example, of urban origin,^39,40^ from the oil and gas industry,^41^ and from sugar cane vinasse.^42^

Among the publications available in the literature, many studies simultaneously address hydrogen production and wastewater treatment,^43,44^ technical assessments of water sources,^45,46^ and evaluations of the economic impacts of using effluents in the electrolysis process.^46^ In terms of application scale, most studies are conducted at the bench scale or remain grid-connected,^47,48^ and many focus on biological optimization in microbial electrolysis cells.^49,50^

Despite the existence of extensive literature addressing different topics, there remains a gap in studies that provide a technical evaluation of a green hydrogen production system powered exclusively by a hybrid renewable matrix (solar and wind), using industrial effluents as feedstock, and applied to the regional context of northeastern Brazil.

Therefore, this study is positioned as a direct response to the gap identified above, detailing its unique configuration and providing essential technical foundations for future economic analyses and real-scale implementation, thereby filling a strategic gap in the national literature on green hydrogen.

In this context, the objective of this study is to simulate the dynamics of an off-grid green hydrogen production system, based on the electrolysis of effluents from a home appliance manufacturing industry located in northeastern Brazil, and powered by solar photovoltaic and wind energy (Off-Grid GH_2_PS). This study aims to address the following questions: What technological capacity is required for the system components to meet industrial energy demands? How does renewable energy flow occur within this off-grid configuration? How does water flow within the water-conditioning subsystem of the proposed model? What is the dynamic behavior of the production, storage, and consumption of electricity and GH_2_? What is the positive environmental impact, in terms of CO_2_eq, resulting from the implementation of this system?

The analysis of the Off-Grid GH_2_PS system dynamics employs a logical methodology. First, the study location and available renewable energy resources are presented. Next, it describes the current energy and input demands of the industry (the current energy scenario) and the system designed to partially meet these demands (the proposed energy scenario). Finally, the simulation configurations used to answer the study questions are detailed.

## Methodology

### Study Location and Energy Resources Data

This case study focused on a company that manufactures household appliances, such as stoves, refrigerators, freezers, and air conditioners. The company is located in the metropolitan region of Fortaleza, in the State of Ceará, Brazil, specifically in the industrial district within the urban area, as shown in Figure 1a.

**Figure 1:**
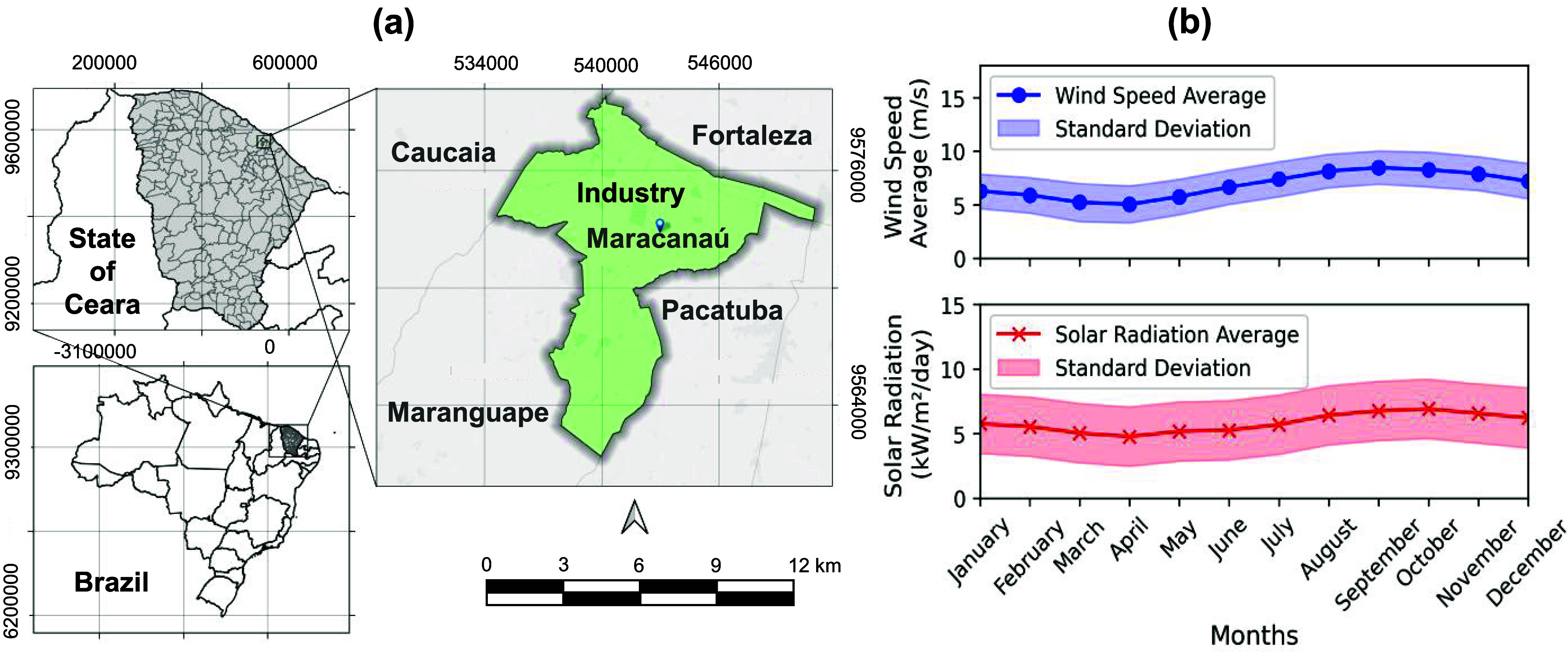
(a) Geographical location of the studied industry. Metropolitan region of Fortaleza, Ceará, Brazil, situated in the industrial district of the urban area of Maracanaú. (b) Monthly averages of global horizontal solar radiation (kW/m^2^/day) and wind speed (m/s) were used to simulate the proposed energy scenario.

Simulations for the proposed scenario used energy resource data from the POWER (Prediction of Worldwide Energy Resources) Project at NASA’s Langley Research Center.^51^ Additionally, the A305 meteorological station, which is located near the studied industry, in Fortaleza (coordinates: −3.815701, −38.537792), was used as a reference when accessing the POWER project database.

The simulations used an average monthly global horizontal radiation based on a 22-year time series (1983–2005). The average wind speed at 50 m above ground level comes from a 30-year time series (1984–2013).^51^ Figure 1b shows the monthly average daily data for solar radiation and wind speed. Figure 1b presents the annual behavior of solar irradiance and wind speed at the study site. The annual solar irradiance is 6.86 ± 1.23 kWh/m^2^/day, whereas the mean wind speed is 5.84 ± 0.72 m/s.

### Baseline Energy Scenario and Water Profile

The studied industry is supplied with electricity and natural gas delivered by local utilities, and it performs its own wastewater treatment (Figure 2a). The industrial wastewater treatment plant (WWTP) is the primary electricity consumer, with an average daily consumption of 28.73 ± 4.92 kWh at high voltage (Figure 2b). Additionally, the industry uses 174,923.6 m^3^ of natural gas per month, supplied by the regional gas company, to generate heat through industrial gas burners used in ovens and dryers at the production facility. Figure 2b shows the monthly natural gas consumption profile, with an average daily consumption of 5642.70 ± 2772.54 m^3^. Furthermore, the industry treats approximately 6075 m^3^ of wastewater per month. This wastewater is transferred to the regional water and sewage company under a pre-established agreement and in accordance with the applicable environmental regulations.^52^

**Figure 2:**
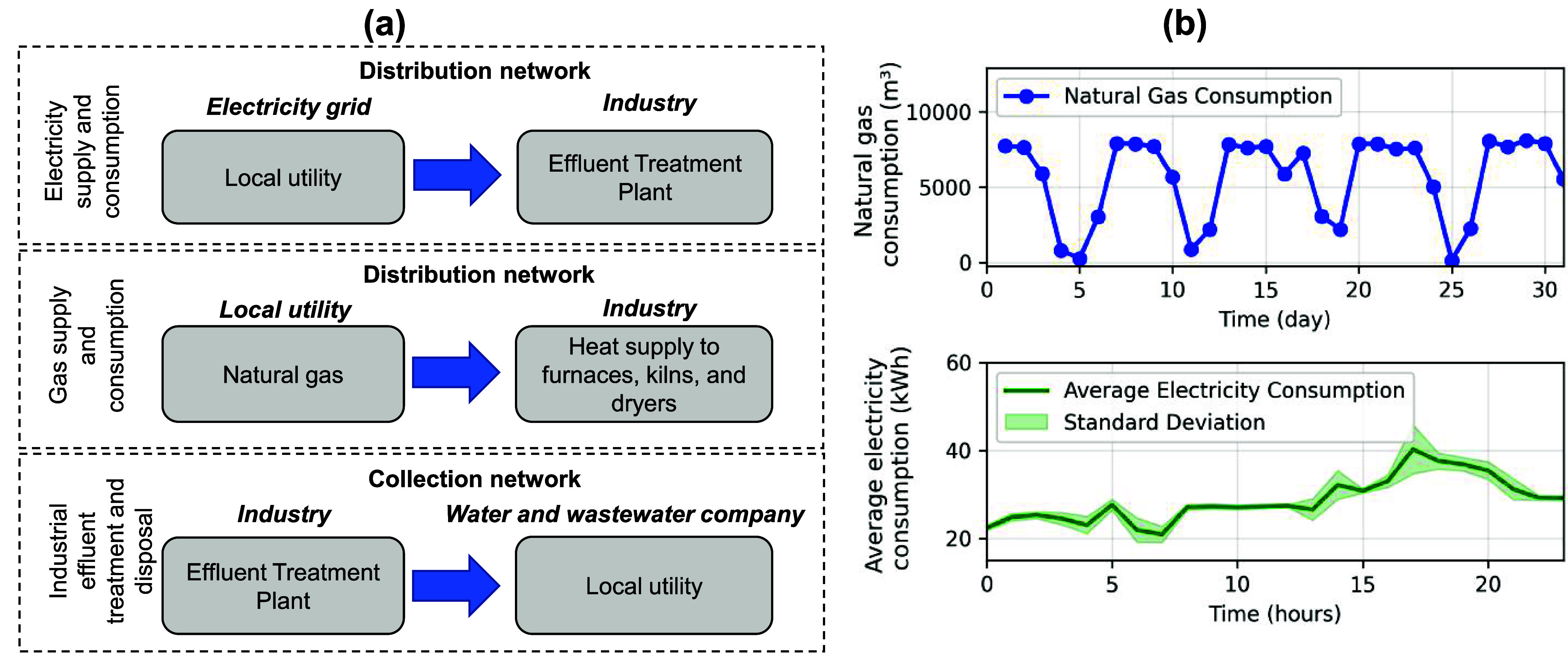
(a) Current scenario of the industry includes the supply and consumption of electricity and natural gas, and the provision of wastewater to the regional water and sewage company. (b) Energy consumption (kWh) and natural gas consumption (m^3^/day) of the Industrial Wastewater Treatment Plant (WWTP).

The industrial facility provided hourly electricity consumption data for a single representative month from which the average hourly load profile was calculated. This mean profile was replicated across all 12 months of the year to construct the annual electricity demand time series. For natural gas consumption, only daily values were available for the same period. These data were converted into an average hourly profile and likewise assumed constant throughout the year. This procedure ensured consistency between the data sets and compatibility with the temporal resolution required by the simulation model. Similar approaches—using representative average profiles when long-term, high-resolution data sets are not available—are widely reported in hybrid energy system modeling.^53,54^

Although the measured electricity and natural gas demand exhibited limited intraday variability, no data were provided to characterize seasonal or operational fluctuations over longer periods. Therefore, a constant hourly profile was adopted as a proxy for annual industrial behavior. This assumption avoids the introduction of synthetic variability and is consistent with accepted modeling practices in the literature, in which representative average profiles are used to preserve the long-term energy balance in the absence of complete data sets.^53,54^

### Proposed Energy Scenario

The proposed energy scenario (see Figure 3) is divided into four segments: (1) utilization of renewable natural resources and renewable electricity generation; (2) energy storage and conditioning; (3) GH_2_ production and water and gas conditioning; and, finally, (4) GH_2_ utilization and industrial wastewater sourcing.

**Figure 3:**
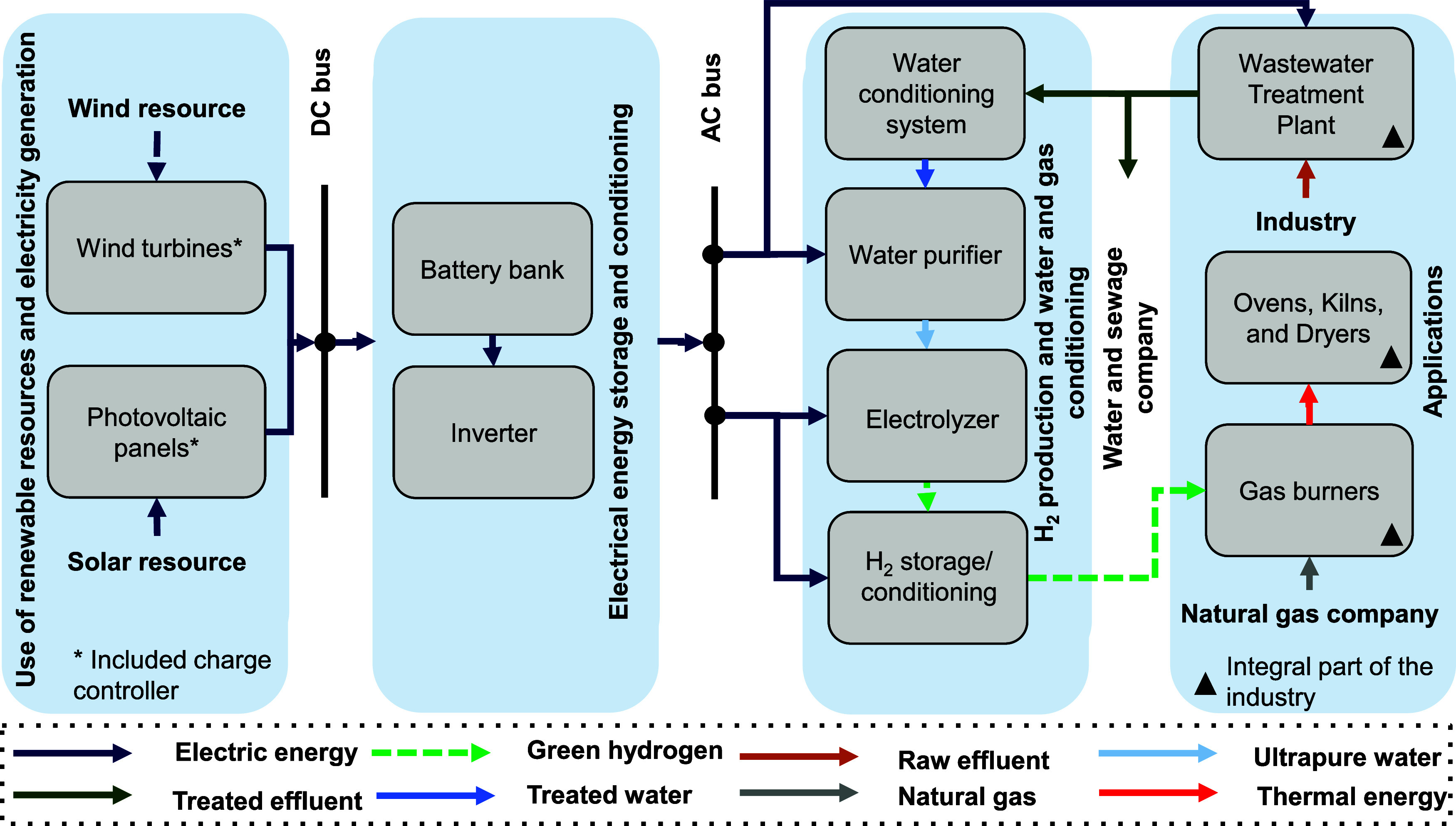
Proposed energy scenario: Utilization of renewable resources and electricity production; energy storage and conditioning; H_2_ production and water and gas conditioning; and applications.

The first segment of the system leverages local wind and solar resources to generate renewable electricity to meet industrial WWTP demand. The second segment stores energy in batteries and redirects it to alternating current loads via an inverter when it is necessary. The third segment involves producing, conditioning, and storing of GH_2_ using PEMEL, storage tanks, and water treatment for electrolysis. The fourth segment outlines using GH_2_ for industrial process heating and reusing wastewater produced by industry for GH_2_ production. The proposed system uses natural gas mixtures containing 10% hydrogen, equivalent to 17,492.36 m^3^ H_2_/month, via modern gas burners that can handle up to 20% hydrogen in the mixture.^55^ Furthermore, the study compares pollutant emissions from both scenarios in a simplified way.

The water-conditioning and purification subsystem was modeled externally through spreadsheet calculations using Microsoft Excel, exclusively to determine the mass balances associated with effluent pretreatment and purification. The energy consumption associated with pumps, microalgae reactors, and purification equipment was incorporated into the main simulation in HOMER Pro^Ⓡ^ as electrical loads, thereby ensuring that the energy demand of the water-conditioning and purification subsystem remained fully accounted for within the overall system energy balance and hydrogen-production balance.

This modular, multisoftware strategy is widely adopted in the literature. Dadak et al.^56^ used Aspen HYSYS for process-level thermodynamic modeling and HOMER Pro^Ⓡ^ for renewable-supply optimization, while Ekpotu et al.^57^ combined HOMER, Aspen HYSYS, and MATLAB/Simulink for the design of a solar-powered PEM electrolysis system. Therefore, the decomposition adopted in this work is technically consistent with modeling practices reported in the literature, ensuring the transparent and traceable quantification of both energy and water flows.

The adopted conditioning system includes biological treatment with microalgae, followed by a purification process using reverse osmosis and electro-deionization. The biological treatment is designed to process 1.26 m^3^/day and consumes 19.28 kWh/day (or 15.30 kWh/m^3^), which is three times higher than the base system designed by Kumar et al.^58^ The purification process uses reverse osmosis and electro-deionization technology from Jonsson e Mässgård^59^ to produces Type I water (less than 1.0 μSiemens/cm), as defined by ASTM Standard N° 1193–99,^60^ with a flow rate of 5 m^3^/h and an energy consumption of 1.534 kWh/m^3^. The volume of ultrapure water feeding the electrolyzer was calculated using the eq 1:

1 $$V_{\mathrm{water},\;\mathrm{electrolyzer}}=\left(M_{\mathrm{hydrogen produced}}\cdot M_{\mathrm{water}‐\mathrm{hydrogen ratio}}\right)$$

where *V*_water, electrolyzer_ is the volume of ultrapure water feeding the electrolyzer (m^3^/month), *M*_hydrogen produced_ is the mass of H_2_ produced during the simulation, and *M*_water‑hydrogen ratio_ is the mass ratio of water to hydrogen.

The simulations adopted a ratio of 9 kg of ultrapure water to produce 1 kg of H_2_.^61^ The corresponding volume of wastewater simulations was determined using the eq 2:

2 $$V_{\mathrm{wastewater}}=V_{\mathrm{water},\mathrm{electrolyzer}}\cdot \left(1.02\cdot 2.50\cdot 1.20\right)$$

where *V*_wastewater_ is the volume of effluent entering the water-conditioning system (m^3^/month), *V*_water,electrolyzer_ is the volume of water feeding the electrolyzer (m^3^/month), 1.02 represents the losses during the transport of wastewater from the WWTP to the conditioning system and from the purifier to the electrolyzer, and 2.50 refers to the recovery rate of the purification system, associated with reverse osmosis. Recovery was between 40 and 60% for large systems (m^3^/month),^62^ and 1.20 represents the losses in the microalgae system, with 80% efficiency.^58^

### Simulation Configuration

This study used HOMER Pro^Ⓡ^ academic (x64) software developed by the National Renewable Energy Laboratory (NREL) (in homerenergy.com). The selection of the HOMER Pro software for conducting the simulations is based on a set of technical and methodological factors that make it particularly suitable for the analysis of hybrid renewable energy systems.^63^ As highlighted in the literature,^53^ among the software tools used in this field, only a limited number simultaneously offer comprehensive features for technical and economic analysis, optimization, and sensitivity assessment. Among these tools are HOMER, iHOGA, SOLSIM, and TRNSYS, which incorporate most of these functionalities.

The choice of HOMER Pro^Ⓡ^ is justified by its combination of functional breadth, extensive recognition in the literature, the robustness of its optimization algorithms, and its ability to generate integrated technical and economic results—characteristics that are essential to the scope of this study.^64^

In addition, HOMER Pro^Ⓡ^ presents several advantages that make it a robust tool for off-grid and on-grid system analyses. The software features a user-friendly interface, a broad database of commercial components, consolidated mechanisms for importing climate data, and allows for detailed hour-by-hour energy balance modeling and comprehensive economic evaluation over the project’s lifetime.^63^ Another decisive feature is its integrated simulation–optimization–sensitivity analysis process, which enables users to identify not only the technical performance of the system but also the most economically viable configuration, considering uncertainty scenarios such as variations in equipment prices, tariffs, fuel costs, and macroeconomic parameters.^65,66^

The optimization methodology implemented in HOMER Pro^Ⓡ^ is based on defining capacity limits for each component to be optimized, followed by the initial sampling of candidate configurations within this range. From these preliminary simulations, the software constructs an approximate model of the net present cost (NPC) behavior, which guides the search toward the most promising system configurations. Subsequently, the focus factor is applied to progressively narrow the search space, concentrating simulations in the most efficient regions. The component sizes are then refined iteratively until the design accuracy and NPC convergence criteria are satisfied. This approach enables HOMER Pro^Ⓡ^ to identify the optimal system configuration while requiring significantly fewer simulations compared to traditional exhaustive-search methods.^63^

The software employs both technical and economic parameters, regardless of whether the study involves exclusively a technical assessment of a given energy system, as is the case in this work. These parameters are used by the software to calculate the real discount or interest rate over the assumed project lifetime, which in this study was set at 25 years. Among the financial parameters adopted in the simulation is the nominal basic interest rate, which in Brazil corresponds to the SELIC rate (13.8%).^67^ Another parameter included is the expected inflation rate (4.18%), calculated based on the Broad Consumer Price Index (IPCA).^68^

To perform the simulations, optimization parameters and constraints were defined to regulate the behavior of the analyzed systems. These parameters include the time step size, which is 1 h and totals 8760 h per year; the option to use multiple generators; and the option to use up to two types of wind turbines. Other considerations, classified as alerts, are not detailed because they are irrelevant to the results.

Restrictions in the software adjust simulations to the system’s dynamics and constrain its behavior in specific situations. These restrictions include: (1) allowing the use of more economical equipment with reduced capacities, which limits the annual capacity shortage to up to 10%; (2) enabling the use of nonrenewable energy sources and ensuring a minimum 10% fraction of nonrenewable energy; and (3) allocating 10% of the energy produced as an operational reserve to manage sudden variations in load or renewable energy production and ensure system stability.

In HOMER Pro^Ⓡ^ software, it was also necessary to adjust the settings related to the optimization algorithm used in the simulations. These adjustable parameters include the maximum number of simulations per optimization (1000); the system design accuracy (0.01), which defines the acceptable relative error in decision variables, such as equipment capacities; the net present cost (NPC) accuracy (0.01), which is related to convergence in the calculation of the system’s total cost; and the focus factor (50), which limits the search space for the system component capacities.

In addition to the outputs provided by the technical analysis, the simulations quantified the emissions of pollutants from the energy generation processes. Penalties were applied to these pollutants to incorporate the environmental impacts generated by the system’s deployment and operation. The pollutants considered include CO_2_, CO, unburned hydrocarbons, particulate matter, SO_2_, and NO_*x*_.

The emissions accounted for are generated by three different sources: electricity production in generators, thermal energy generation in burners or boilers, and electricity consumption from the local grid, as described in NREL.^63^ These emissions were calculated according to the source of pollution.

For generators, burners, or reformers, the annual emission of a given pollutant (kg/year) was determined using the eq 3:

3 $$E_{\mathrm{annual},\mathrm{pollutant}}=f_{\mathrm{emission},\mathrm{pollutant}}\cdot C_{\mathrm{annual},\mathrm{total},\mathrm{fuel}}$$

where *f*_emission,pollutant_ is the emission factor of a given pollutant (g/m^3^) and *C*_annual,total,fuel_ is the annual fuel consumption (m^3^/year).

For electricity consumption from the grid, the annual emission of a given pollutant (kg/year) was determined using the eq 4:

4 $$E_{\mathrm{annual},\mathrm{pollutant}}=f_{\mathrm{emission},\mathrm{pollutant}}\cdot E_{\mathrm{annual},\mathrm{purchased},\mathrm{grid}}$$

where *f*_emission,pollutant_ is the emission factor of a given pollutant (g/m^3^) and *E*_annual,purchased,grid_ is the annual electricity purchased from the local grid.

In the context of emission quantification, the current simulated scenario relies on the local grid for its electricity supply, which is predominantly hydroelectric and represents 57% of Brazil’s internal electricity supply.^15^ Although hydroelectricity is renewable, it also produces pollutant emissions. According to Fearnside,^69^ these emissions can reach approximately 279.04 gCO_2_/kWh and 38.34 gCH_4_/kWh. Therefore, this emission source was considered in the simulations.

In the proposed scenario, the use of natural gas mixtures containing 10% H_2_ was intended to reduce pollutant emissions, as the combustion of such a mixture in industrial burners produces approximately 26% less CO and NOx than the combustion of 100% natural gas.^70^ In the simulations, the mixture was considered to emit 110 mg of CO/m^3^ and 100 mg of NOx/m^3^, while pure natural gas was considered to emit 148 mg of CO/m^3^ and 135 mg of NOx/m^3^. Regarding the unburned hydrocarbons, which are predominantly composed of CH_4_, 36.80 g CH_4_/m^3^ of pollutant emissions was adopted.^71^

As described in Table 1, the software received technical data for each piece of equipment used in the system, including capacity, useful life, and efficiency. It is important to note that the adopted equipment consists of commercially available solutions, which ensures the credibility and realism of the simulations.

**Table 1:** Capacity, Efficiency, Durability, or Lifetime Data of the Components of the Simulated Off-Grid GH_2_PS System

Components	Capacity (kW, kg*, unid**, L/h***)	Efficiency (%)	Lifetime (years)
Photovoltaic module	0.33 ^72^	17 ^72^	25 ^72^
Wind turbine	1 ^72^	96 ^72^	20 ^73^
Battery	1**^72^	98 ^74^	12 ^74^
Inverter/rectifier	1 ^75^	92 ^75^	20 ^75^
Eletrolyzer	1 ^76^	52 ^79^	15 ^77^
H_2_ tank	1*^76^		30 ^78^
Water purifier	5000***^59^	40 ^62^	variable^59^
Microalgae treatment	52.50***^58^	80 ^58^	variable^58^

The capacity values of the components listed in Table 1 were reduced from the values provided by the manufacturer: the wind turbine and the inverter by 10 times, and the battery by 5 times. This reduction was necessary to improve the performance of the optimization algorithm. It is important to note that the data referring to the burners were not included in the simulations because they are already part of the existing industrial installations; therefore, the current simulated model does not consider the burners’ combustion process.

The software requires capacity value ranges for each system component in order to calculate the optimal configuration of the proposed system for simulations. The capacity values were set to ranges of 200 to 1400 kW and kg for the electrolyzer and fuel tank. Meanwhile, the software optimizer adjusted the capacity of the other system components based on energy and hydrogen demand. Additionally, the model assumes that electricity and heat consumption and production do not vary throughout the system’s lifespan in the study scenarios. Table 2 presents the technical, economic, and simulation parameters used in the study.

**Table 2:** Technical, Economic, and Simulation Parameters Used in the Study

**Category**	**Parameter/information**	**Value/setting**	**Description/Notes**
**Economic data**	nominal base rate	Selic: 13.80%	used as the nominal interest rate in Brazil
	expected inflation rate	4.18% (IPCA)	used to calculate the real discount rate
	project lifetime	25 years	horizon for technical and economic assessments
**General simulation data**	software used	HOMER Pro^Ⓡ^ (academic version)	integrated technical, economic, and environmental analysis
	time step size	1 h (8760 h/year)	temporal resolution of the simulations
	multiple generators	allowed	enables the use of more than one generator type
	wind turbine types	up to 2 types	software-imposed limitation
**Model constraints**	annual capacity shortage allowed	up to 10%	allows the use of smaller and more economical components
	minimum nonrenewable energy fraction	10%	ensures a minimum participation of nonrenewable sources
	operating reserve	10% of the produced energy	ensures stability against load or renewable fluctuations
**Optimization algorithm parameters**	maximum simulations per optimization	1000	maximum number per optimization cycle
	system design accuracy	0.01	acceptable relative error for decision variables
	NPC accuracy	0.01	convergence criterion for net present cost calculations
	focus factor	50	restricts the search space around promising regions
**Emissions and environmental impacts**	pollutants considered	CO_2_, CO, unburned HC, PM, SO_2_, NOx	based on NREL methodology
	emission sources	generators, burners/boilers, grid electricity	three distinct emission origins
**Component capacity adjustments**	wind turbine derating	1/10 of the manufacturer’s rating	applied to improve optimization performance
	inverter derating	1/10 of the manufacturer’s rating	same rationale
	battery derating	1/5 of the manufacturer’s rating	same rationale
	burner data	not included	burners are part of the existing facility; combustion not modeled
**External components**	water treatment	modeled externally (excel)	HOMER does not include a water treatment module
**Capacity ranges for optimization**	electrolyzer capacity	200–1400 kW	search range defined by the user
	hydrogen tank capacity	200–1400 kg	same search range
	other system components	automatically adjusted	based on energy and hydrogen demand
**General model assumptions**	consumption and production profiles	constant for over 25 years	no seasonal variation or equipment degradation considered

### Assumptions and Scope of the Study

The study focuses on the technical analysis of the energy, water, and environmental dynamics of an off-grid green hydrogen production system based on the electrolysis of industrial effluents powered by renewable energy (solar and wind). The geographical scope is limited to an industry located in Northeastern Brazil, whose climatic and operational characteristics may not be fully generalizable to other regions.

Technically, the study includes only the modeling of solar and wind power generation, battery-based electricity storage, hydrogen storage in a pressurized tank, hydrogen production via a PEM electrolyzer, and the use of hydrogen blended with natural gas. Economic and life-cycle assessments, broader socioenvironmental impacts, and indirect emissions are not considered. Environmentally, the study is limited to estimating avoided CO_2_eq emissions resulting from the partial replacement of natural gas and grid electricity without including other impact categories.

The study also covers the water balance involving the flows of wastewater and processed water within the water-conditioning subsystem but does not include detailed analyses of water quality or hydrological impacts in the study region or the effects of interferences on the overall process efficiency or on the effectiveness of wastewater treatment, as these topics are not the focus of the manuscript. Nevertheless, the reader may consult the literature for information on water and wastewater purification technologies intended for electrolysis, such as the review published by Becker et al.^80^ Regarding the application of microalgae in wastewater treatment with a focus on biofuel production, the reader may refer to the study developed by de Oliveira et al.^81^

The simulations of the Off-Grid GH_2_PS system were developed based on assumptions that allow the system’s technical behavior to be represented under typical operating conditions. It was assumed that the solar radiation and wind speed profiles used adequately reflect the climatic regime of Northeastern Brazil for the study site without accounting for atypical events.

It was also assumed that the industry’s demands for electricity, natural gas, and water remain constant over the project period, with only seasonal or annual operational variations. The quality of the effluent treated by the water-conditioning subsystem within the Off-Grid GH_2_PS was considered adequate for supplying the PEM electrolyzer without requiring additional purification processes. Furthermore, all components—photovoltaic panels, wind turbines, inverters, batteries, hydrogen storage units, and the electrolyzer—were assumed to operate at constant efficiencies, with the degradation scheduled within the analysis period.

The model further assumes that all hydrogen produced is used in the NG–H_2_ blend (10–90%) for heat generation via combustion, without considering losses due to compression, transport, or pressure variations in the storage system. Aspects related to reaction selectivity, faradaic efficiency, electrode degradation pathways, or the occurrence of secondary reactions were not analyzed in the study, as they are intrinsic to the operation of the commercial electrolyzer adopted in the simulations.

Finally, it is important to note that the proposed modeled energy system did not undergo direct experimental validation nor benchmark validation due to the absence of a real system at the industrial facilities adopted in the case study and the lack of a significantly similar system reported in the literature. Nevertheless, the model possesses conceptual and theoretical validation, as it was developed using the widely recognized HOMER Pro^Ⓡ^ software, developed by the National Renewable Energy Laboratory (NREL) (available at homerenergy.com), which is based on mathematical equations, physical laws, and theoretical relationships consolidated in the literature and has been adopted in numerous publications across various scientific journals.

## Results and Discussion

### System Architecture and Energy Balance

The Off-Grid GH_2_ production system was simulated to meet the industrial energy demands described in the proposed scenario. The capacities of the wind and solar photovoltaic generation sources and the electrolyzer were defined as 169, 712, and 500 kW, respectively. The battery bank, hydrogen tank, and inverter were sized at 182 kWh, 800 kg, and 144 kW. These values were defined by the software optimizer to minimize the system’s net present cost, which is not discussed in this article. The optimization followed the standard HOMER Pro^Ⓡ^ lowest-cost algorithm, which iteratively evaluates thousands of feasible combinations of component capabilities.^63^

The capacity of the energy generation and hydrogen production technologies depends on local renewable resources. The simulations yielded capacity factors of 48.90% for wind energy, 18.80% for solar photovoltaics, and 31.10% for hydrogen production. These results consist of data released by the Brazilian National Electric System Operator for the same study region (the Ceará coastline) and evaluation period. According to the ONS,^8^ the wind and solar capacity factors are 49 and 27%, respectively. However, the capacity factor for hydrogen production depends on the configuration and dynamics of each system under study. Therefore, its value cannot be used directly for comparison purposes to other systems in the literature.

Although direct comparison is difficult, some decentralized and semipilot systems reported in the literature provide a useful reference. Rahimi and Eicker^82^ analyzed decentralized wastewater treatment units based on microbial electrolysis cells coupled with renewable electricity, and showed that hydrogen production is strongly constrained by the intermittent availability of both wastewater and renewable power, resulting in a relatively modest number of full-load operating hours in decentralized configurations. Heidrich et al.^83^ evaluated a 100-L pilot-scale microbial electrolysis cell (MEC) fed with domestic wastewater over 12 months and reported an average hydrogen production of 0.60 L/day, with 48.70% recovery of the electrical energy input, illustrating the typical performance of semipilot bioelectrochemical systems treating real effluent.

In contrast, Holmes-Gentle et al.,^84^ using a pilot plant with solar concentrators and kilowatt-scale electrolyzers, reported operation under more stable conditions, allowing greater subcomponent utilization, although limited to daytime operation. Taken together, these studies highlight that the hydrogen production capacity factor of 31.10% obtained for the Off-Grid GH_2_PS is consistent with values expected for decentralized hydrogen-production systems constrained by local renewable resources.

It is also important to note that the capacity of photovoltaic generation is 4.2 times greater than that of wind generation due to the difference in the operating time between the two sources. The capacity factor of photovoltaic generation is 2.6 times lower than that of wind generation, which explains this difference. Photovoltaic generation only operates 49.50% of the time due to the limitation of an average of 11.80 h of solar incidence per day, while wind generation operates 90% of the time because it is present throughout the day, according to NASA’s POWER database for the region.^51^

The local availability of natural resources also affected the capacity of the energy generation and hydrogen storage subsystems. This allowed for a reduction in the capacities of the battery bank and hydrogen system, taking advantage of periods of abundant energy to generate and store resources. According to Yang et al.,^85^ a battery bank with reduced capacity compensates for minimal differences between energy demand and generation, a concept applicable to the current study. Similarly, the availability of energy resources enabled a reduction in the capacity of the hydrogen subsystem, thereby increasing the storage capacity. Higher storage capacity enables the electrolyzer to use energy during periods of abundance and store it for use during periods of low availability.^86^

A Sankey diagram was developed (Figure 4) to represent the simulated Off-Grid GH_2_PS in terms of energy flow from the generation source to the loads.^87^ The energy flow of Off-Grid GH_2_PS highlights the relationship between the available primary energy and the fraction effectively converted to useful services. The annual availability of solar and wind energy totals 8,129,331.43 kWh/year, of which 1,899,503.73 kWh/year are converted into electricity, resulting in an overall primary conversion efficiency of 23.36%. Most of the primary energy (approximately 76.7%) remains unused, mainly due to the low photovoltaic conversion efficiency and the Betz limit, which constrains the maximum recoverable wind energy.

**Figure 4:**
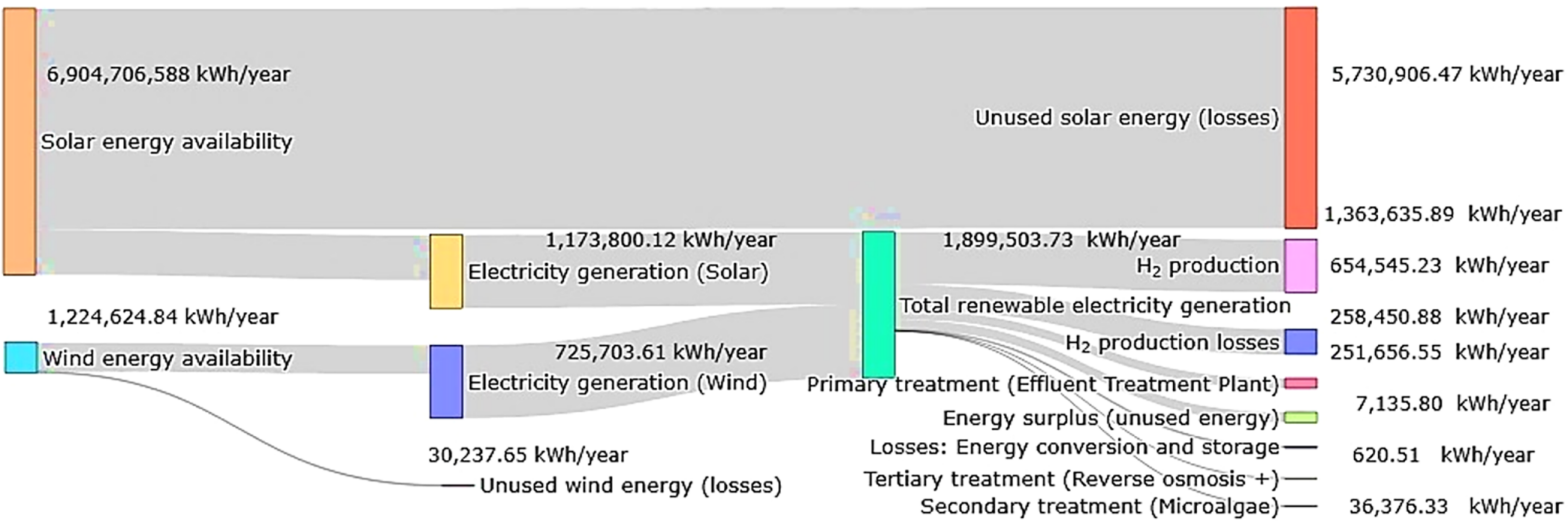
Simplified energy balance for the proposed energy scenario.

Of the total electricity generated, 87.69% is directed to end uses—hydrogen production and industrial effluent treatment—while 12.31% corresponds to surplus energy (251,656.55 kWh/year) or internal losses associated with conversion and storage processes. Additionally, such losses may originate from the electricity consumption of auxiliary processes (e.g., pumping, mixing, control), rather than exclusively from electrolysis, which reduces the total fraction of electricity converted directly into hydrogen.^82^ In the case of hydrogen production, losses vary around 52% for a PEMEL electrolyzer,^79^ whereas an AEMEL unit can reach efficiencies of up to 62.50%, and SOEC or AEL technologies may achieve 84 and 66%, respectively.^88–90^

The percentage of useful energy utilized in the proposed scenario is consistent with that in the literature. For example, Clemens et al.^91^ simulated a large-scale wind–photovoltaic–electrolysis system with underground hydrogen storage and showed, through a Sankey diagram, that about 84% of the generated electrical energy can be directed to hydrogen production when the electrolyzer is properly sized relative to the installed renewable capacity, while the remainder corresponds mainly to grid exports and internal losses.

Hydrogen production constitutes the main demand of the system, consuming 1,363,635.89 kWh/year, which represents 71.79% of the generated electricity and 81.87% of the end-use consumption. Effluent treatment, which comprises primary, secondary, and tertiary stages (microalgae and purification), jointly uses 302,963.01 kWh/year, equivalent to 15.90% of the electricity generated. This occurs because, according to Eurowater,^61^ 2.20 kWh is required to purify one cubic meter of treated effluent. However, the simulations showed that the system under study consumes 16.58 kWh, likely because the microalgae treatment step accounts for 92% of the energy consumption of the tertiary treatment process.

Thus, the system exhibits an overall useful efficiency of 20.49%, defined as the ratio between the energy effectively used by the system (1,665,598.90 kWh/year) and the annual available primary energy. This indicates that although energy capture presents technological limitations, the electricity generated is utilized with high efficiency by the internal processes.

### Renewable Power Systems: Variability, Storage, and Demand Matching

Understanding the behavior of solar incidence and wind speed in the study region is essential to energy production, which is directly related to the availability of local energy resources. According to the simulation data, solar incidence peaks in October (6.92 kWh/m^2^/day) and is lowest in April (4.77 kWh/m^2^/day). Wind speed is at its maximum in September (8.49 m/s) and at its minimum in April (5.04 m/s), which influences wind energy production. These data suggest that the simulated Off-Grid GH_2_PS system is balanced and resilient. Natural resources complement each other, optimizing energy production throughout the year.^92,93^

Solar production peaks in October at 115,604.87 kWh, with an annual average production of 97,816.68 ± 11,202.73 kWh, while wind production also peaks in October at 72,626.96 kWh, with an annual average of 60,472.64 ± 10,912.89 kWh. The system’s total renewable energy production is 1,906,391.82 kWh/year. Data show that solar and wind energy production complement each other throughout the day, with wind energy becoming the primary source at night. Solar energy represents approximately 62% of the total production. Combining solar and wind energy enhances reliability and availability, reducing intermittency. This is supported by the work of Lave and Ellis^94^ and Bubalo et al.^92^ Significant performance gains were observed after adopting the hybrid system proposed by Srivastava.^93^

As discussed earlier, energy production is associated with the loads consumed by the simulated Off-Grid GH_2_PS, including the Effluent Treatment Plant (ETP), the electrolyzer, and the water-conditioning system (SCA) (Figure 5a). ETP energy consumption ranged from 19,305.16 kWh in February to 21,373.57 kWh in subsequent months, indicating low seasonal variability. Similarly, the SCA exhibited stable consumption, fluctuating slightly from 577.35 kWh in February to 658.80 kWh in several subsequent months.

**Figure 5:**
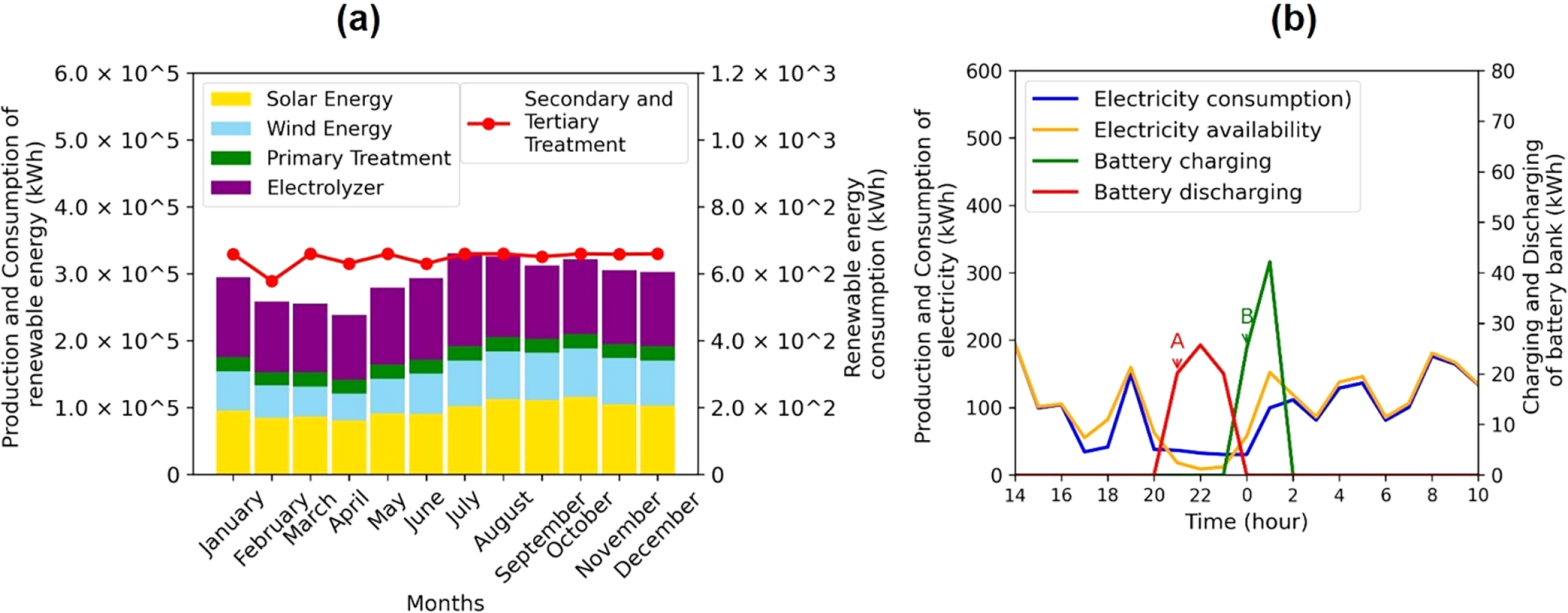
(a) Renewable energy production from wind and photovoltaic solar sources and monthly renewable electricity consumption from the ETP, electrolyzer, and water-conditioning subsystems (kWh); (b) dynamics of renewable energy production and consumption (kWh) and dynamics of renewable energy storage (kWh), including lithium battery charging/discharging.

In contrast, the electrolyzer exhibited significant seasonal variation, with consumption ranging from a low of 96,730.89 kWh in April to a high of 138,577.72 kWh in July. This variation reflects the influence of seasonal factors, likely including renewable energy availability, on hydrogen production.^95^ The annual average consumption for these loads is 251,657 ± 5.02 kWh for the ETP, 1,363,635 ± 171.94 kWh for the electrolyzer, and 7756.3 ± 0.43 kWh for the SCA. The electrolyzer is the largest consumer, representing 84% of the total consumption, followed by the ETP (15.50%) and the SCA (0.48%).

In addition to energy production and consumption, the system uses batteries for energy storage, which increases the system’s reliability, stability, and efficiency, especially in off-grid systems without transmission and distribution losses.^96,97^ Lithium batteries offer advantages such as flexibility in energy storage and greater integration between different renewable sources.^98^ Other benefits include high energy density, a long lifespan, efficient charging and discharging, and a low self-discharge rate.^99^

Energy storage is crucial when production exceeds demand, allowing batteries to charge. It is also important when production is insufficient, which enables the release of stored energy. The battery bank has a capacity of 182.40 kWh; however, only 91.20 kWh is used because the discharge depth is limited to 50% to prolong the battery life.^99^ A total of 23,964 and 23,395 kWh of energy was stored and delivered to the loads, representing less than 1.47% of the total load consumption. As shown in Figure 5b, this stored energy is accessed when demand exceeds the level of production (Point A). As soon as production meets demand, the battery bank ceases supplying energy to the loads and enters charging mode (Point B).

Point A: Consumption exceeds energy production (battery discharge). Point B: Consumption is lower than energy production (battery charging).

### Green Hydrogen Pathways: Generation, Storage, and End-Use Dynamics

Various methods exist for using hydrogen generated through electrolysis, including generating electricity via fuel cells and generating heat through the combustion of the blend in burners.^13,14^ In the proposed scenario, the industry adopted the combustion of hydrogen mixed with natural gas, storing the hydrogen for use as needed due to its ability to be stored long-term with minimal maintenance, despite the specialized and costly infrastructure.^100,101^

The hydrogen–natural gas mixture presents challenges related to transportation, distribution, and final use, primarily due to infrastructure issues. The addition of hydrogen can decrease the mixture’s calorific value and Wobbe index, as well as require an increase in volumetric flow. This may demand larger pipelines or higher operating pressures.^14,100,101^

In the proposed scenario, the industry used a mixture containing 10% hydrogen by volume relative to the amount of natural gas consumed (2,099,083.08 m^3^/year). This resulted in a 3% reduction in the volume of natural gas, in agreement with the findings described by Sorgulu et al.^102^ While adding hydrogen increases the blend’s calorific value, it reduces its volumetric calorific value. This results in a 7% decrease in usable energy compared with pure natural gas. To offset this reduction in energy, the simulation incorporated a 6.5% increase in the blend’s volumetric flow, reaching 2,246,018.896 m^3^/year, which is higher than the industry′s baseline volume consumption.^102,103^

The gas mixture provided 18,828,069.94 kWh/year of energy, which is 1,417,166.554 kWh/year less than the energy consumed by pure natural gas (20,245,236.49 kWh/year). This decrease affects the time required for industrial processes to heat, such as water heating, which may take up to 13.41% longer with the gas mixture.^102,103^ To compensate for this difference, Dzurňák et al.^14^ state that the gas mixture consumption should increase by around 6.7%, in line with the increase in the mixture’s volumetric flow.

The simulation results show an annual production of 17,975.88 kg, with monthly averages of 1497.99 ± 136.02 kg. Consumption is 17,262.20 kg per year, while storage reaches 5383.20 kg, with monthly averages of 1438.52 and 448.60 kg, respectively. As Figure 6a shows, production and storage fluctuate throughout the year in response to demand and renewable energy availability. This behavior is consistent with renewable-electrolyzer integrated systems, where production varies with energy availability.^96,98^

**Figure 6:**
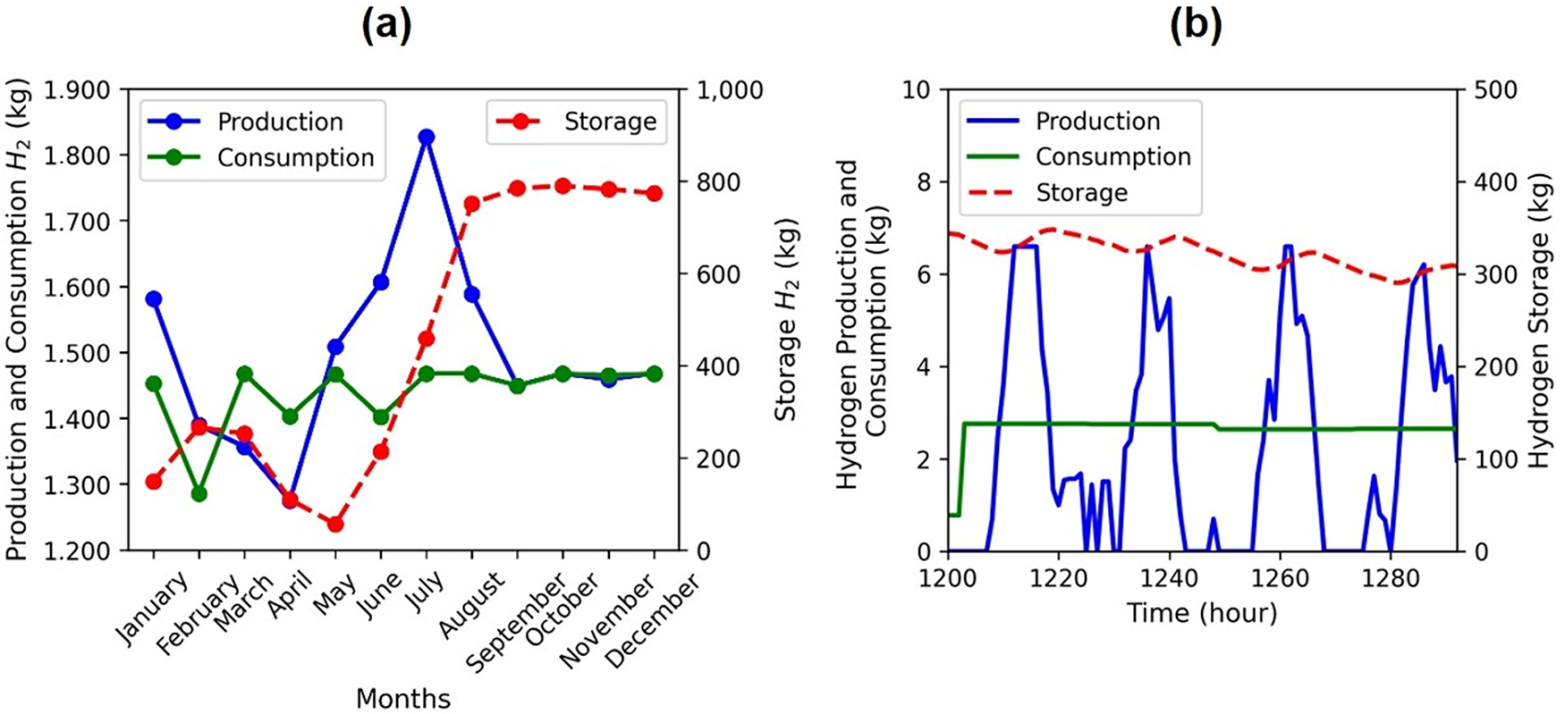
Monthly (a) and hourly (b) production, consumption, and storage of hydrogen (kg) in the proposed scenario.

During the first two quarters, production drops by ∼20%, followed by an increase of ∼40% in the next quarter due to increased energy generation. This increase brings the storage hydrogen to the system’s 800 kg capacity limit. For the remainder of the year, production decreases by ∼20% due to the high level of hydrogen in storage. These supplies meet demand without significant fluctuations until the end of the year.

Analysis of the proposed scenario shows that storage is the key to meeting demand during periods of low production, as illustrated in Figure 6b. Storage provides the system with flexibility and resilience, allowing adjustments in stored hydrogen levels according to consumption.^96–98^ Therefore, the integration of renewable energy with efficient storage management is essential for ensuring a continuous and reliable energy supply that meets the needs of the proposed system.

According to the literature (Table 3), the specific energy consumption for hydrogen production varies depending on the type of electrolyzer and system configuration. Systems based on PEMEL generally show values between 53 and 54.6 kWh/kg of H_2_,^12,28^ while AEL systems report consumption around 51.90 kWh/kg of H_2_.^24^ On the other hand, SOEC can operate with lower energy demands, reaching 42 kWh/kg of H_2_.^20^ However, some studies, particularly those involving complex pretreatments or unconventional configurations, report significantly higher consumption, reaching up to 401.35 kWh/kg of H_2_.^26^ In the proposed scenario, the specific energy consumption was 75.90 kWh/kg of H_2_. This higher value is primarily the result of the low utilization rate (a capacity factor of 31.1%) and frequent operation under partial load (an average of 2 kg of H_2_/h out of a maximum of 6.59 kg of H_2_/h). These conditions are caused by the intermittency of the renewable energy source.^93,94^ These conditions compromise the electrolyzer’s efficiency (43.90%) and justify the higher specific consumption.^19,85^

**Table 3:** Specific Energy and Effluent Consumption in Green Hydrogen Production Using Different Electrolyzers and Renewable Energy Sources

**Renewable energy**	**Effluent**	**Tertiary treatment**	**Electrolyzer**	**kg of effluent/kg H**_**2**_	**kWh/kg H**_**2**_	**Refs**
Thermal and photovoltaic solar power and wind power	Wastewater treatment plant	Ultrafiltration (UF) process, an ultraviolet (UV) disinfection, and membrane distillation powered by thermal solar energy	PEMEL	18.54^a^	53.71^b^	^27^
Photovoltaic solar power and wind power	Wastewater treatment plant	Single-stage RO, recovery rate of 50%, and TDS concentration in the feed equal to 20.6 ppm	AEL	18.20^c^	51.90^d^	^24^
Photovoltaic solar power	Wastewater treatment plant	Not specified	PEMEL	27.74^e^	53^f^	^28^
Photovoltaic solar power and wind power	Wastewater treatment plant	Tertiary filtration (without details)	SOEC	38.2–103.1^g^	42^h^	^20^
Photovoltaic solar	NaOH and effluents from granite cuttings employing conventional loom (CLG)	Reverse osmosis for NaOH	Own design with characteristics of an alkaline electrolyzer	NaOH 127.22	NaOH 67.41^j^	^26^
				CLG 232.25^i^	CLG 401.35	
Photovoltaic solar	Flue gas desulfurization wastewater	The pressurized wastewater is routed to a drum of the hot flue gas duct for evaporation. A portion of the wastewater that is vaporized in the drum is distilled	PEMEL	198.10^k^	54.50^l^	^22^
Photovoltaic solar	Sewage treatment plant	Tertiary treatment (without details)	PEMEL	18^m^	54.60^n^	^12^

a Assumed 1 kg of effluent/h and 0.6 Nm^3^ H_2_/h (≈0.0539 kg H_2_/h [0.0899 kg/Nm^3^]).

b Based on LHV of 33.30 kWh/kg H_2_ and 62% electrolyzer efficiency.

c Based on 9.1 kg of water/kg H_2_ (electrolyzer datasheet) and 50% recovery by reverse osmosis.

d Electrolyzer datasheet.

e Based on 260 kg/h of water (datasheet) and 50% recovery by reverse osmosis.

f Electrolyzer datasheet.

g Estimated from two simulated effluent streams with yields of 26.20 and 9.70 kg H_2_/m^3^.

h Includes total net electrical and thermal energy for SOEC system operation.

i Determined by the ratio between total electrical energy supplied and the mass of H_2_ produced (volume measured using CNTP density [0.0899 g/L]).

j Obtained by dividing the reactor volume (6.87 cm^3^) by the mass of H_2_ generated in each test.

k Determined by the ratio between 69.81 L/h of effluent and the hourly mass of H_2_ produced (0.3524 kg/h [3.92 Nm^3^/h × 0.0899 kg/Nm^3^]).

l Based on LHV of 33.30 kWh/kg H_2_ and 61% electrolyzer efficiency.

m Obtained by the ratio between 9800 m^3^ of effluent per day and the production of 544,444 kg H_2_ per day.

n Electrolyzer datasheet.

### Water Conditioning Used in the Proposed Scenario

The production of GH_2_ via water electrolysis, powered by renewable energy, requires high-quality water. The necessary specifications vary depending on the technology used. Typically, these requirements follow the ASTM D1193-91 classification, which categorizes water as type I, II, III, or IV based on its physicochemical properties.^60^ Major electrolyzer manufacturers specify the necessary water quality in their catalogs. Nel ASA recommends types I and II water for PEMEL and AEL technologies; Fuel Cell Energy suggests type I for solid oxide electrolyzers; and Enapter allows type IV but recommends type III or II for its AEMEL.^88,89,104^ Unlike other technologies, MEC (Microbial Electrolytic Cell) electrolyzers use wastewater or fermentable or nonfermentable substrates.^105^

The growing demand for purified water for electrolysis affects the energy consumption of the electrolyzers. According to Eurowater,^61^ the electrolysis process consumes 5000 kWh/m^3^ of ultrapure water. In this study, however, consumption was higher at 5800 kWh; this is greater than values reported by electrolyzer manufacturers: 4824.46 kWh/m^3^ for solid oxide;^89^ 5405.41 kWh/m^3^ for AEL;^106^ and 5305.26 kWh/m^3^ for AEM.^88^ Additionally, the volume of water consumed varies by electrolyzer technology. Values include 0.17 L/h/kW for PEMEL;^79^ 0.18 L/h/kW for AEL;^106^ 0.19 L/h/kW for AEM;^88^ and 0.21 L/h/kW for solid oxide.^89^

Using the water quality and volume requirements of different technologies, we analyze the water flow in the simulated Off-Grid GH_2_PS system. In the proposed scenario, hydrogen production uses some of the effluent sent to the regional water and sewage company. Figure 7a illustrates this process. It shows that 486.81 m^3^/year of effluent undergoes treatment for GH_2_ production, of which 162.27 m^3^/year reaches the electrolyzer. By comparison, 324.54 m^3^/year becomes waste after being treated by microalgae and the water purification subsystem (tertiary treatment). According to the technical report prepared by the University of Queensland and Monash University, which presents strategies for integrating large-scale wastewater treatment facilities with water electrolysis systems, although additional treatment stages increase specific energy consumption and introduce nonrecoverable water losses, these losses remain manageable when the influent is tertiary-treated municipal wastewater. This reinforces the importance of effluent quality in minimizing the water footprint of hydrogen production.^107^

**Figure 7:**
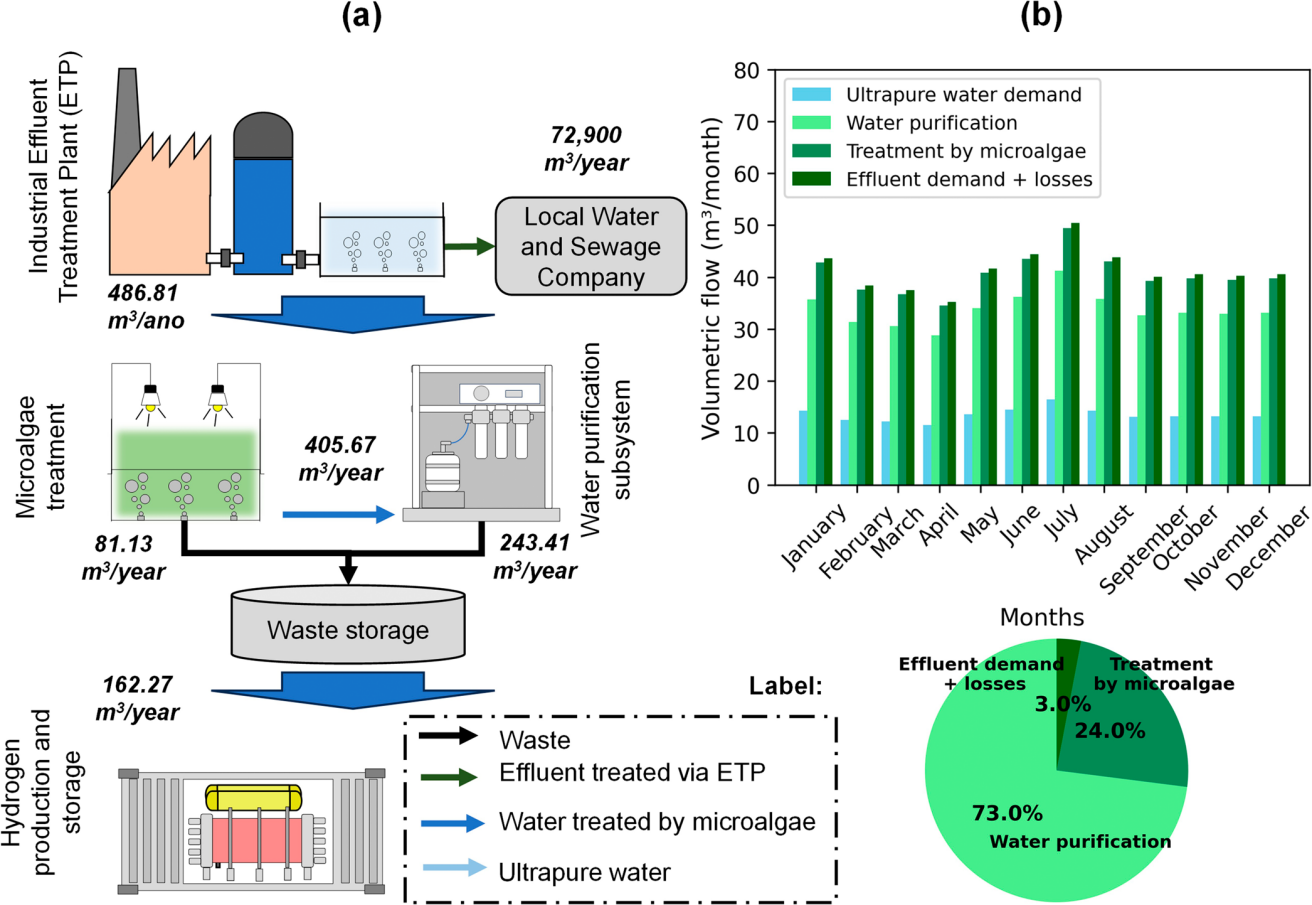
(a) Water flow across the components of the water-conditioning subsystem in the simulated Off-Grid GH_2_PS system; (b) volumetric flow and water losses throughout the water-conditioning system.

The water-conditioning process involves several stages and generates monthly variations in consumption that reflect seasonal and operational influences. Figure 7b shows how water consumption changes over time. It highlights that April and July have the lowest and highest effluent consumption, respectively, at 35.22 and 50.46 m^3^/month. These variations are proportional to the availability of renewable electricity (solar and wind), consequently influencing hydrogen production.^19,20^ Additionally, the demand for effluent and ultrapure water varies throughout the year, with average values of 41.38 ± 3.76 and 13.52 ± 1.23 m^3^/month, respectively. The difference in these volumes is due to water losses in the water-conditioning subsystem. About 73% of the water is lost in the purification stage, 24% is lost in the microalgae treatment stage, and 3% is lost in the water transport between stages. This data is crucial for developing strategies that minimize water loss in the GH_2_ production process.

Manufacturers of electrolyzers report only the volume of treated water required to produce 1 kg of H_2_, ignoring the total amount of water used, including that lost during the process. This value varies between 11.11 kgH_2_O/h for PEMEL,^79^ 9.46 kgH_2_O/h for AEL,^106^ 9.44 kgH_2_O/h for solid oxide,^89^ and 10.04 kgH_2_O/h for AEMEL.^88^ The simulations assumed that 15 kg of water produces 1 kg of H_2_ since industrial effluent has characteristics similar to fresh water from rivers and lakes.^61^ Recent reviews^108,109^ on hydrogen production from wastewater emphasize that the overall water demand depends not only on the stoichiometric requirement of electrolysis but also on the type of effluent, the intensity of pre- and post-treatment processes, and the configuration of the electrochemical system in use.

According to the literature, effluent consumption per kilogram of H_2_ varies considerably depending on the type of wastewater, the applied treatment, and the electrolysis system configuration, as shown in Table 3. The lowest levels of consumption were around 18 kg/kg H_2_, as observed in processes using treated municipal effluent.^12,24^ In contrast, the highest levels reached 232.25 kg/kg H_2_, as in the case of industrial effluents from granite cutting using Conventional Loom Granite (CLG).^26^ Using more complex effluents or those with a high dissolved solid content tends to significantly increase water demand to lower conversion efficiency and the need for more intensive treatment. For example, flue gas desulfurization produces effluent with a consumption level of 198.10 kg/kg H_2_.^22^ However, the simulations indicated that 27.54 kg of effluent is required to produce 1 kg of GH_2_. This difference is attributed to losses in the water-conditioning system, as previously described.

Pilot-scale microbial electrolysis-based systems, such as the one studied by Heidrich et al.,^83^ operate with raw or lightly pretreated effluent and therefore do not require the production of ultrapure water, which avoids the large purification losses observed in the Off-Grid GH_2_PS. However, these systems typically achieve limited hydrogen yields and only partial recovery of the supplied electrical energy, with average energy recovery below 50%, and they often focus primarily on wastewater treatment rather than maximizing hydrogen production. In contrast, the Off-Grid GH_2_PS integrates advanced tertiary treatment and ultrapure water production to meet electrolyzer specifications, which increases energy consumption and water losses in the conditioning subsystem but enables higher hydrogen productivity and compliance with industrial water quality requirements. This comparison highlights that both the type and quality of the effluent and the chosen electrolysis technology strongly influence the balance between water demand, energy consumption, and hydrogen yield.^80,82^

### Environmental Impact of the Proposed Implementation Scenario

The study analyzed emissions from fuel gas consumption in both the current and proposed scenarios and from electricity consumption from the local power grid in the current scenario. According to the literature, when the hydrogen percentage in the natural gas mixture reaches 20%, CO_2_ emissions decreased by 6–7% without the need to alter the combustion infrastructure.^110,111^ Furthermore, the NO_*x*_ emissions vary according to the percentage of hydrogen. For instance, Ozturk et al.^70^ found that 10% H_2_ in the mixture caused a 26% reduction in NO_*x*_ emissions, whereas 20% H_2_ resulted in a 20% increase.

Burning of natural gas produces CO_2_ and NO_*x*_ emissions, but adding hydrogen decreases them. The hydrogen-gas natural mixture reduces CO_2_ emissions from 148 mg/m^3^ to 110 mg/m^3^ and NO_*x*_ emissions from 135 mg/m^3^ to 100 mg/m^3^.^70^ Additionally, natural gas combustion generates unburned hydrocarbons, including methane, at a rate of 36.80 mg/m^3^.^71^ Currently, gas and electricity from the local grid, which are predominantly hydroelectric,^15^ contribute to emissions at values of 279.04 gCO_2_/kWh and 38.34 gCH_4_/kWh.^69^

A quantitative analysis of emissions revealed that CO_2_ emissions account for most of the total emissions in both the current (91.67%) and proposed (98.10%) scenarios (see Table 4). According to Korb et al.,^112^ the presence of hydrogen in the natural gas mixture resulted in a significant reduction in CH_4_ emissions (from 6.42 to 0.35%) and improved combustion performance. NO_*x*_ emissions also decreased, from 1.91 to 1.54%.^70^ Overall, the proposed scenario showed a 10.99% reduction in pollutant emissions, equivalent to approximately 465.47 tCO_2eq_/year. This reduction is due to the substitution of grid electricity with renewable sources and the reduction in natural gas consumption.

**Table 4:** Greenhouse Gas Emissions (CO_2_, CO, CH_4_, and NO_*x*_) Generated by the Current and Proposed Energy Scenarios

	**Current scenario**		**Proposed scenario**	
**Pollutant**	**Quantity** (kg/year**)**	**tCO**_**2eq**_ ^a^	**Quantity** (kg/year**)**	**tCO**_**2eq**_ ^a^
Carbon dioxide	3,881,647.25	3881.65	3,697,272.04	3697.27
Carbon monoxide	207.76		149.78	
Unburned hydrocarbons	9700.19	271.61	50.11	13.28
Nitrogen oxides	305.21	80.88	219.30	58.12
Total	3,891,860.41	4234.13	3,697,691.23	3768.67

a The pollutants CO_2_, CH_4_, and NO_*x*_ have global warming potentials of 1, 28, and 265, respectively, as described in the Assessment Report of the Intergovernmental Panel on Climate Change.^117^

Recent literature highlights the importance of evaluating the sustainability characteristics of renewable energy production systems, especially the transition toward circular economy concepts and energy-sustainable configurations, in which water reuse, energy recovery, and emission reduction are jointly assessed.^113^ In the hydrogen sector, the concept of sustainability and life-cycle assessment has been increasingly used to compare production pathways and to identify options with lower environmental burdens, including those that integrate effluent treatment and renewable hydrogen generation.^108,114^

Although the present study does not conduct a complete sustainability assessment, such as a life-cycle analysis, its structure inherently incorporates key sustainability principles by using industrial effluents as feedstock, operating exclusively with renewable energy sources, and quantifying the CO_2_eq emissions avoided by the proposed off-grid GH_2_ production system.

Considering that CO_2_ is the primary pollutant, it is crucial to adopt scenarios such as the one proposed. The increase in greenhouse gas emissions, in particular, CO_2_, is a global concern. CO_2_ accounts for 64% of emissions, followed by CH_4_ at 19%.^115^ Furthermore, CO_2_ emissions contribute to ocean acidification, which negatively affects marine ecosystems.^116^ Reducing these emissions is crucial to mitigating climate change, which reinforces the importance of investing in low-carbon technologies.

### Limitations of the Study

The study presents some limitations inherent to the data set and the modeling environment. The absence of a real system required the use of technical parameters provided by equipment manufacturers and theoretical values found in the literature, hindering direct validation of the simulated model. The lack of finer temporal resolution profiles for electricity and natural gas consumption limits the ability to capture operational transients and dynamic variations within the system. The assumption of fixed component efficiencies prevents the representation of degradation, overload, thermal effects, or failures, resulting in an invariant operational dynamic. This occurs due to the exploratory nature of the simulations, especially when reliable degradation data for all subsystems are not available or are highly variable in the literature. In addition, the emissions assessment is limited to avoided direct emissions and does not include emissions associated with manufacturing, transportation, maintenance, or disposal of equipment.

The use of constant hourly load profiles across the simulated year represents an inherent limitation resulting from the restricted availability of industrial energy consumption data. Consequently, the model may under-represent peak loads, operational transients, and seasonal dynamics that could influence the optimal sizing of components such as the battery bank and the electrolyzer. Nonetheless, for the purposes of this study—focused on annual energy balance, system integration, and techno-environmental performance—representative average profiles offer sufficient fidelity, as supported by previous modeling studies that adopt similar simplifications when long-term data sets are unavailable.^53,54^

Modeling the water-conditioning and purification subsystem externally and decoupled from the dynamic behavior of hydrogen production represents a methodological simplification and a limitation of the study. Furthermore, the water-conditioning subsystem does not introduce additional GHG emissions because all electricity consumed by the water treatment and water purification processes is supplied entirely by renewable sources (solar and wind). In this context, at first glance, performing a full LCA of the energy pathway may appear to be a limitation; however, its inclusion would not significantly alter the results, since the environmental impact is driven not by energy consumption but by water use and water losses, which are already quantified and discussed in detail in the manuscript.

These limitations may affect the absolute accuracy of the results, although they do not compromise their usefulness for comparative and exploratory purposes relative to the literature.

## Conclusion

This study evaluated the technical feasibility of an Off-Grid GH_2_ production system (Off-Grid GH_2_PS), powered by renewable energy, and using industrial effluents as feedstock for electrolysis. Implementing the Off-grid GH_2_PS in the analyzed industrial context could reduce pollutant emissions by up to 11%, equivalent to approximately 465.47 tCO_2_eq/year. This reduction would be primarily achieved by replacing grid electricity with renewable sources and reducing natural gas consumption. Furthermore, simulations indicated an energy consumption of 75.9 kWh per kg of H_2_ produced and 5800 kWh per m^3^ of ultrapure water consumed. The water-to-hydrogen conversion rate required 27.54 kg of effluent per kilogram of hydrogen. These results suggest that the system has the potential to partially meet industrial energy demands through a sustainable, decentralized approach.

The article makes scientific and technical contributions by proposing a scalable, off-grid solution aligned with the global energy transition. This solution is capable of attracting strategic investments, especially in regions with abundant renewable resources and industrial effluents. However, the study faced several challenges, including a lack of high-resolution consumption data and operational data from a real water-conditioning system. Additionally, software limitations required the use of a spreadsheet to model certain subsystems. These factors resulted in simplified dynamic modeling, which affected the accuracy of the expected performance gains.

Future research should prioritize the incorporation of high-resolution time-series load profiles as well as the inclusion of temporal variations in industrial demand in order to improve the accuracy of long-term simulations. In addition, the use of biogas from wastewater treatment as a complementary renewable energy source is recommended as it may reduce the required storage capacity and enhance overall system efficiency. Furthermore, future studies should integrate component degradation models for the system elements present in the proposed energy scenario, enabling more realistic projections of their performance over the operational lifetime. Finally, fully dynamic coupling between the water treatment subsystem and the energy–hydrogen production chain, together with a detailed life-cycle assessment (including embodied emissions of the treatment infrastructure), would allow a more comprehensive evaluation of the system’s sustainability under variable and long-term operating conditions.

Referring to the practical implications of the study, the results demonstrate the feasibility for industrial facilities to adopt distributed hydrogen production via electrolysis, integrating off-grid renewable resources, and using their own effluents as feedstock. Moreover, they provide practical guidance for the partial replacement of natural gas with green hydrogen, reducing CO_2_ emissions, lowering effluent disposal costs, and improving industrial energy security. The developed model also serves as a preliminary planning tool for industries seeking to evaluate and replicate similar systems.

Finally, it is important to note that global warming caused by environmental pollution is reversible as long as the energy transition occurs through global decarbonization as soon as possible. This includes adopting renewable energy sources, such as GH_2_.

## Symbols, Abbreviations, and Nomenclatures

ABH_2_: Associação Brasileira de Hidrogênio (*Brazilian Hydrogen Association*)
AEMEL: Anion Exchange embrane Electrolyzer
ANEEL: Agência Nacional de Energia Elétrica (*National Electric Energy Agency*)
ASTM: American Society for Testing and Materials
CAGECE: Companhia de Água e Esgoto do Ceará (*Water and Sewage Company of Ceará*)
CNTP: Normal Temperature (0 °C or 273.15 K) and Pressure Conditions (1 atm or 101.33 kPa)
COEMA: Conselho Estadual do Meio Ambiente (*State Environmental Council*)
CO_2_: Carbon dioxide
CO: Carbon monoxide
CPH_2_: Clean power hydrogen
*E*_renewable electricity consumption_: Potential revenue associated with renewable electricity consumption by the WWTP (USD)
*E*_annual,pollutant_: Annual emission of a given pollutant (kg/year)
*E*_annual,purchased,grid_: Annual electricity purchased from the local grid (kWh/year)
*E*_avoided,emissions_: Avoided emissions – difference between current and proposed energy scenario emissions (kg/year)
*E*_def_: deferrable electrical load (kWh)
*E*_electricity consumption reduction_: Savings from reduced electricity grid consumption (USD)
*E*_emissions produced_: Emissions per kg of H_2_ produced (kg CO_2_eq/kg H_2_)
*E*_equivalent,carbon_: Carbon equivalent emissions (kg/year)
*f*_emission,pollutant_: Emission factor of a given pollutant (g/m^3^ or g/kWh, depending on the case)
GH_2_: green Hydrogen
HC: Unburned hydrocarbons
H_2_LAC: Plataforma para el Desarrollo del Hidrógeno Verde en América Latina y el Caribe
HOMER: Hybrid Optimization Model for Electric Renewables
IBGE: Instituto Brasileiro de Geografia e Estatística (*Brazilian Institute of Geography and Statistics*)
IEA: International Energy Agency
IPCA: Índice Nacional de Preços ao Consumidor Amplo (*Broad National Consumer Price Index*)
LHV: Lower Heating Value
Matlab: Matrix Laboratory
MEC: Microbial electrolytic cells
M_hydrogen produced_: Mass of green H_2_ produced annually (kg H_2_/ano)
M_water‑hydrogen ratio_: Mass ratio of water to hydrogen (kg of water per kg of H_2_)
NASA: National Aeronautics and Space Administration
Nox: Nitrogen oxides
NPC: Net present cost
NREL: National Renewable Energy Laboratory
Off-grid GH_2_PS: Green Hydrogen Production System Powered by Off-grid Renewable Energy through the Electrolysis of Industrial Effluents
PEMEL: Proton exchange membrane electrolyzer
PHV: Portal Hidrogênio Verde *(Green Hydrogen Portal)*
PM: Particulate matter
POWER: Prediction of Worldwide Energy Resources
SOEC: Solid oxide electrolysis cell
SO_2_: Sulfur dioxide
UF: Ultrafiltration
UV: Ultraviolet
*V*_wastewater_: Volume of effluent entering the water-conditioning system (m^3^/month)
*V*_water, electrolyzer_: Volume of ultrapure water feeding the electrolyzer (m^3^/month)
WWTP: Wastewater treatment plant

## References

[1] UNFCCC , United Nations Framework Convention on Climate Change. The Paris Agreement: What is the Paris Agreement? United Nations Framework Convention on Climate Change, 2024. https://unfccc.int/process-and-meetings/the-paris-agreement (accessed Sept 13, 2024).

[2] Griffiths S., Sovacool B. K., Kim J., Bazilian M., Uratani J. M. (2021). Industrial
decarbonization via hydrogen: A critical and systematic review of
developments, socio-technical systems and policy options. Energy Res. Soc. Sci..

[3] Dincer I. (2012). Green methods
for hydrogen production. Int. J. Hydrogen Energy.

[4] Nikolaidis P., Poullikkas A. (2017). A comparative overview of hydrogen production processes. Renewable and Sustainable Energy Rev..

[5] Shiva
Kumar S., Himabindu V. (2019). Hydrogen production by PEM water
electrolysis – A review. Mater. Sci.
Energy Technol..

[6] Kim J. H., Hansora D., Sharma P., Jang J.-W., Lee J. S. (2019). Toward
practical solar hydrogen production – an artificial photosynthetic
leaf-to-farm challenge. Chem. Soc. Rev..

[7] Ishaq H., Dincer I., Crawford C. (2022). A review on
hydrogen production and
utilization: Challenges and opportunities. Int.
J. Hydrogen Energy.

[8] Zeng Q., An W., Peng D., Liu Q., Zhang X., Ge H., Liu H. (2025). Research Progress in
Photocatalytic-Coupled Microbial Electrochemical
Technology in Wastewater Treatment. Catalysts.

[9] Landman A., Halabi R., Dias P., Dotan H., Mehlmann A., Shter G., Halabi M., Naserladeen O., Mendes A., Grader G. S., Rothschild A. (2020). Decoupled
Photoelectrochemical Water Splitting System for Centralized Hydrogen
Production. arXiv.

[10] Bora L. V., Bora N. (2025). Photoelectrocatalytic Water Splitting
for Efficient Hydrogen Production:
A Strategic Review. Fuel.

[11] Andrade, A. R. ; Castro, A. S. ; Reginatto, V. Sistemas Bioeletroquímicos: Célula Eletrolítica Microbiana para a Produção de Hidrogênio Revista Virtual de Química 2024; Vol. 16 1 10.21577/1984-6835.20230055.

[12] Barghash H., Al Farsi A., Okedu K. E., Al-Wahaibi B. M. (2022). Cost benefit
analysis for green hydrogen production from treated effluent: The
case study of Oman. Front. Bioeng. Biotechnol..

[13] Bedakhanian A., Assareh E. (2024). Exploring an innovative
approach to hydrogen generation
for fuel cell energy production by integrating a dual organic Rankine
system with an absorption chiller powered by geothermal energy. Energy Nexus.

[14] Dzurňák R., Jablonský G., Pauerová K., Eliaš R. (2024). Enriching
Natural Gas with Hydrogen: Implications for Burner Operation. Eng. Proc..

[15] EPE, Empresa de Pesquisa Energética . Balanço Energético Nacional. Empresa de Pesquisa Energética (EPE), 2022. https://www.epe.gov.br/pt/publicacoes-dados-abertos/publicacoes/balanco-energetico-nacional-2022 (accessed June 20, 2023).

[16] IEA - International Energy Agency . Energy Statistics Data Browser – Data Tools - IEA 2022. https://www.iea.org/data-and-statistics/data-tools/energy-statistics-data-browser?country=WORLD&fuel=Energy+supply&indicator=ElecGenByFuel. (accessed May 10, 2023).

[17] ONS, Operador Nacional do Sistema Elétrico . Resultados da Operação: Geração e fator de capacidade médios mensais. 2022. https://www.ons.org.br/Paginas/resultados-da-operacao/historico-da-operacao/geracao-fator-capacidade-medios-mensais.aspx (accessed May 3, 2024).

[18] ANEEL, Agência Nacional de Energia Elétrica . SIGA - Sistema de Informações de Geração da ANEEL. 2023. https://dadosabertos.aneel.gov.br/dataset/siga-sistema-de-informacoes-de-geracao-da-aneel (accessed June 14, 2023).

[19] Simoes S.
G., Catarino J., Picado A., Lopes T. F., Di Berardino S., Amorim F., Gírio F., Rangel C. M., Leão P. T. (2021). Water availability
and water usage solutions for electrolysis in hydrogen production. J. Cleaner Prod..

[20] Maddaloni M., Marchionni M., Abbá A., Mascia M., Tola V., Carpanese M. P., Bertanza G., Artioli N. (2023). Exploring the Viability
of Utilizing Treated Wastewater as a Sustainable Water Resource for
Green Hydrogen Generation Using Solid Oxide Electrolysis Cells (SOECs). Water.

[21] Das S., Peter S. C. (2024). Green Hydrogen from
Wastewater – A Dual Crisis
Resolution. Energy Fuels.

[22] Fakourian S., Alizadeh M. (2023). Hydrogen Generation
from the Wastewater of Power Plants
via an Integrated Photovoltaic and Electrolyzer System: A Pilot-Scale
Study. Energy Fuels.

[23] Cvetković S. M., Radoičić T. K., Novaković J. G., Kovačević V., Lopičić Z. R., Adamović V., Kijevčanin M.
L. (2022). Renewable hydrogen production
perspective in Serbia via biogas generated from food processing wastewaters. J. Cleaner Prod..

[24] Campana P. E., Mainardis M., Moretti A., Cottes M. (2021). 100% renewable wastewater
treatment plants: Techno-economic assessment using a modelling and
optimization approach. Energy Convers. Manage..

[25] Jolaoso L. A., Asadi J., Duan C., Kazempoor P. (2023). A novel green
hydrogen production using water-energy nexus framework. Energy Convers. Manage..

[26] Marques F. C., Silva J. C. M., Libardi C. P., de Carvalho R. R., Sequine G. F., Valane G. M. (2020). Hydrogen production
by photovoltaic-electrolysis
using aqueous waste from ornamental stones industries. Renewable Energy..

[27] Marzo E., Galí A., Lefevre B., Bouchy L., Vidal A., Cortina J. L., Fabre A. (2012). Hydrogen and Oxygen
Production Using Wastewater Effluent Treated with Ultra-Filtration
and Membrane Distillation (Greenlysis). Procedia
Eng..

[28] Donald R., Boulaire F., Love J. G. (2023). Contribution to Net Zero Emissions
of Integrating Hydrogen Production in Wastewater Treatment Plants. J. Environ. Manage..

[29] AQUATECH . Developing Hydrogen From Wastewater – Anglian & Element 2 Partnership Leads Uk Efforts. AQUATECH, June 21, 2023. https://www.aquatechtrade.com/news/water-treatment/generate-green-hydrogen-with-wastewater (accessed Aug 21, 2023).

[30] Biogradlija, B. A. Unlocking the Potential: UK Project Turns Wastewater into Green Hydrogen. Energy News, May 31, 2023. https://energynews.biz (accessed July 17, 2023).

[31] Carroll, D. . Australian developer moves forward with solar-to-hydrogen project. PV magazine Australia, June 13, 2023. https://www.pv-magazine.com/2023/06/13/australian-developer-moves-forward-with-solar-to-hydrogen-project/ (accessed Aug 7, 2023).

[32] Morais, L. Cepsa to fuel green hydrogen plant with recycled wastewater in Spain. Renewables Now, December 20, 2022. https://renewablesnow.com/news/cepsa-to-fuel-green-hydrogen-plant-with-recycled-wastewater-in-spain-808785/ (accessed July 23, 2023).

[33] Himmelstein, S. Plant to produce green hydrogen from reclaimed water. GlobalSpec, July 14, 2023. https://insights.globalspec.com/article/20614/plant-to-produce-green-hydrogen-from-reclaimed-water (accessed July 20, 2023).

[34] CPH2, Clean Power Hydrogen . CPH2 and NI Water, working together to reduce emissions. Clean Power Hydrogen (CPH2), June 15, 2021. https://www.cph2.com/news/cph2-and-ni-water-working-together-to-reduce-emissions/ (accessed June 15, 2023).

[35] PLUG POWER INC . Plug’s California Green Hydrogen Plant Saves Water, Creates New Energy Source. PLUG POWER INC, December 6, 2022. https://www.plugpower.com/plugs-california-green-hydrogen-plant-saves-water-creates-new-energy-source/.

[36] H2LAC . Colombia ya tiene sus primeras moléculas de hidrógeno verde. Plataforma para el desarrollo del hidrógeno verde en latinoamérica y el Caribe (H2LAC), March 22, 2022. https://h2lac.org/noticias/colombia-ya-tiene-sus-primeras-moleculas-de-hidrogeno-verde/ (accessed June 3, 2023).

[37] Serpa, E. Cagece tem toda a água para as usinas do Hidrogênio Verde. Diário do Nordeste, May 24, 2022. https://diariodonordeste.verdesmares.com.br/opiniao/colunistas/egidio-serpa/cagece-tem-toda-a-agua-para-as-usinas-do-hidrogenio-verde-1.3234701 (accessed Aug 22, 2023).

[38] SEINFRA, Secretaria de Infraestrutura . Primeira molécula de Hidrogênio Verde produzida no Brasil é lançada no Ceará. Governo do Estado do Ceará, 2023. https://www.seinfra.ce.gov.br/2023/01/19/primeira-molecula-de-hidrogenio-verde-produzida-no-brasil-e-lancada-no-ceara/ (accessed Feb 7, 2023).

[39] Nadaleti W.
C., Gomes J. P. (2023). Green Hydrogen
Production from Urban Waste Biogas:
An Analysis of the Brazilian Potential and the Process’s Economic
Viability. Renewable Sustain. Energy Rev..

[40] Silva G. O. R. E., Carpanez T. G., Dos Santos C. R., de Paula E. C., Amaral M. C. S. (2024). Biohydrogen Production
from Wastewater: Production
Technologies, Environmental and Economic Aspects. J. Environ. Chem. Eng..

[41] M
de Araujo D., Barbosa Segundo I. D., Cardozo J. C., dos Santos E. V., Martínez-Huitle C. A. (2024). Produced Water Electrolysis
with Simultaneous Green H_2_ Generation: From Wastewater
to the Future of the Energetic Industry. Fuel.

[42] Anzola-Rojas M. D. P., Sánchez F. E., Fuess L. T., Pant D., Zaiat M. (2025). Hydrogen
Production from Fermented Sugarcane Vinasse
and Its Utilization by Biosynthesis Processes in a Single-Chambered
Microbial Electrolysis Cell. Int. J. Hydrogen
Energy..

[43] Alcaraz-Gonzalez V., Rodriguez-Valenzuela G., Gomez-Martinez J. J., Dotto G. L., Flores-Estrella R. A. (2021). Hydrogen
Production Automatic Control in Continuous Microbial Electrolysis
Cells Reactors Used in Wastewater Treatment. J. Environ. Manage..

[44] Sgarbi R., Mandelli C. M., Ruotolo L. A. M. (2025). Integrated Renewable
H_2_ Production and Industrial Wastewater Treatment Using
a Semi-Pilot
Scale Flow Electrolyzer. Chem. Eng. J..

[45] Santana L. O. S., Bispo A. S., Santos G. S., Almeida J. L. G., Pessoa F. L. P. (2023). Water
Sources Evaluation for Green Hydrogen Production: A Case Study in
Brazil. Comput.-Aided Chem. Eng..

[46] Santana, L. O. S. ; dos Santos, G. S. ; Marinho, C. B. ; Almeida, J. ; Pessoa, F. L. P. Evaluation of Different Water Sources for Electrolysis: A Study Case of the Priority Regions for Green Hydrogen Production in the State of Bahia. In Computer-Aided Chemical Engineering; Cavalcanti, T. V. ; Pereira, L. M. ; Silva, A. F. , Eds.; Elsevier: Amsterdam, 2024; Vol. 53, pp 2281–2286 10.1016/B978-0-443-28824-1.50381-1.

[47] Maciel O. S., Caldeira V. P. S., Cardozo J. C., dos Santos E. V., de Araújo D. M. (2025). Sustainable Electrochemical Integrated-Hybrid
Process for Degrading Caffeine and Producing Green Hydrogen. J. Solid State Electrochem.

[48] Monteiro M. K. S., Cardozo J. C., Araújo A. M. D.
M., Quiroz M. A., dos Santos E. V. (2025). Electrochemical Looping Green Hydrogen Production
by Using Water Electrochemically Treated as a Raw Material for the
Electrolyzer. Catalysts.

[49] Kadier A., Wang J., Chandrasekhar K., Abu Hasan H., Ma P.-C. (2022). Performance Optimization of Microbial Electrolysis Cell (MEC) for
Palm Oil Mill Effluent Wastewater Treatment and Sustainable Bio-H_2_ Production. Int. J. Hydrogen Energy.

[50] Grangeiro, L. C. ; de Mello, B. S. ; Rodrigues, B. C. G. ; Sarti, A. ; Dussán, K. J. Dark Fermentation and Principal Routes to Produce Hydrogen. In Materials for Hydrogen Production, Conversion, and Storage; John Wiley & Sons, Ltd, 2023, pp 181–223. 10.1002/9781119829584.ch7 (accessed Dec 1, 2025).

[51] LARC, NASA Langley Research Center . The POWER Project. 2022. https://power.larc.nasa.gov/ (accessed March 31, 2023).

[52] COEMA, Conselho Estadual do Meio Ambiente . RESOLUÇÃO COEMA N^ *o* ^02, de 02 de fevereiro de 2017. 2017. https://www.semace.ce.gov.br/resolucao-2017-coema/ (accessed May 28, 2023).

[53] Sinha S., Chandel S. S. (2014). Review of software
tools for hybrid renewable energy
systems. Renewable Sustainable Energy Rev..

[54] Lambert, T. ; Gilman, P. ; Lilienthal, P. Micropower System Modeling with HOMER. In Integration of Alternative Sources of Energy; Farret, F. A. ; Simões, M. G. ,, Eds.; John Wiley & Sons, Inc.: Hoboken, NJ, USA, 2006, pp 379–418. 10.1002/0471755621.ch15 (accessed Dec 1, 2025).

[55] WEISHAUPT . Os queimadores WeishauptWK (até 32.000 kW). WEISHAUPT, 2019. https://www.weishaupt.com.br/produtos/queimador/queimadores-weishaupt-linha-wk-ate-32000-kw (accessed Oct 4, 2024).

[56] Dadak A., Mousavi S. A., Mehrpooya M., Kasaeian A. (2022). Techno-Economic Investigation
and Dual-Objective Optimization of a Stand-Alone Combined Configuration
for the Generation and Storage of Electricity and Hydrogen Applying
Hybrid Renewable System. Renewable Energy.

[57] Ekpotu W. F., Akintola J. T., Obialor M. C., Udom P. C. (2023). A Solar Energy System
Design for Green Hydrogen Production in South-Western Nigeria, Lagos
State, Using HOMER & ASPEN. Open J. Optim..

[58] Kumar A. K., Sharma S., Dixit G., Shah E., Patel A. (2020). Techno-economic
analysis of microalgae production with simultaneous dairy effluent
treatment using a pilot-scale High Volume V-shape pond system. Renewable Energy.

[59] Jonsson, A. ; Mässgård, H. An Industrial Perspective on Ultrapure Water Production for Electrolysis. Ph.D. Dissertation, Kungliga Tekniska Högskolan, Skolan För Industriell Teknik Och Management, 2021. https://www.diva-portal.org/smash/get/diva2:1575929/FULLTEXT01.pdf (accessed Sept 17, 2023).

[60] ASTM , International. Standard Specification for Reagent Water; ASTM D1193–99e1, 2017. DOI: 10.1520/D1193-99E01.

[61] Eurowater . Water treatment for green hydrogen WHAT YOU NEED TO KNOW. EUROWATER A GRUNDFOS COMPANY, 2022. https://www.eurowater.com/Files/Files/eurowater/Country/International/Leaflets/White-paper_water-treatment-for-hydrogen_EUROWATER.pdf (accessed June 5, 2024).

[62] Stoughton, K. M. ; Duan, X. ; Wendi, E. M. Reverse Osmosis Optimization. U.S. Department of Energy, 2013. http://doi.wiley.com/10.1002/9780470882634 (accessed Feb 10, 2024).

[63] NREL, National Renewable Energy Laboratory . HOMER Pro User Manual V.13. 2019. https://www.homerenergy.com/products/pro/docs/index.html (accessed June 11, 2023).

[64] Kavadias K.
A. (2021). Triantafyllou,
Panagiotis. Hybrid renewable energy systems’ optimization.
A review and extended comparison of the most-used software tools. Energies.

[65] El
Boujdaini L., Jurado F., Mezrhab A., Moussaoui M. A., Vera D. (2023). Cost and Size Optimization of Hybrid Solar and Hydrogen Subsystem
Using HOMER Pro Software. Int. J. Hydrogen Energy..

[66] Suresh V., Muralidhar M., Kiranmayi R. (2020). Modelling and Optimization of an
Off-Grid Hybrid Renewable Energy System for Electrification in Rural
Areas. Energy Rep..

[67] Banco Central do Brasil – BC . Taxa Selic. Governo Federal do Brasil, 2023. https://www.bcb.gov.br/controleinflacao/taxaselic (accessed June 30, 2023).

[68] IBGE , Instituto Brasileiro de geográfica e estatistica. Inflação. Governo Federal do Brasil, 2023. https://ibge.gov.br/explica/inflacao.php (accessed June 23, 2023).

[69] Fearnside P. M. (2002). Greenhouse
Gas Emissions from a Hydroelectric Reservoir (Brazil’s Tucuruí
Dam) and the Energy Policy Implications. Water,
Air, Soil Pollut..

[70] Ozturk M., Sorgulu F., Javani N., Dincer I. (2023). An experimental study
on the environmental impact of hydrogen and natural gas blend burning. Chemosphere.

[71] ERG, Eastern Research Group . Emission Factor Documentation for Ap-42 Section 1.4 Natural Gas Combustion. U.S. Environmental Protection Agency, 1998. https://www.epa.gov/sites/default/files/2020-09/documents/background_document_ap-42_section_1.4_natural_gas_combustion.pdf (accessed Sept 22, 2023).

[72] Intelbras . Módulo Fotovoltaico Policristalino 72 células 330 W - Ems 330p. INTELBRAS, 2022. https://backend.intelbras.com/sites/default/files/2022-03/Datasheet_EMS_330P_Frame_35mm_R1.pdf (accessed Aug 27, 2023).

[73] Aeolos Wind Turbine . Aeolos-V 10kW vertical wind turbine. 2023a. https://www.windturbinestar.com/10kwv-v-aeolos-wind-turbine.html (accessed April 1, 2023).

[74] UNIPOWER . Bateria Lítio Ferro Fosfato - LiFePO4 UPLFP48–100–3U. UNIPOWER, 2023. https://www.neosolar.com.br/amfile/file/download/file/74/product/31161/ (accessed April 18, 2024).

[75] Aeolos Wind Turbine . Vertical-axis small wind turbine AEOLOS-V 10KW. 2023b. [Email] message received by: < sales@windturbinestar.com> (accessed Feb 19, 2023).

[76] Tractebel; Engie; Hinicio . Early Business Cases for H2 In Energy Storage and More Broadly Power To H2 Applications. Fuel Cell And Hydrogen Joint Undertaking, 2017. https://hsweb.hs.uni-hamburg.de/projects/star-formation/hydrogen/P2H_Full_Study_FCHJU.pdf (accessed Sept 2, 2023).

[77] Breitenstein M., Hicks A. (2022). Review and
harmonization of the life cycle global warming impact
of five United States aquaponics systems. Aquacult.
Eng..

[78] BAYO TECH . Fuel Storage Modules. BAYO TECH, 2023. https://bayotech.us/ground-storage-modules/ (accessed Jan 6, 2023).

[79] SWISSGAS, LNI . H2 POWER 10L PEM Hydrogen generator system. LNI SWISSGAS, 2023. https://www.lni-swissgas.eu/en/product/h2-power-10l/ (accessed Aug 12, 2023).

[80] Becker H., Murawski J., Shinde D. V., Stephens I. E. L., Hinds G., Smith G. (2023). Impact of Impurities
on Water Electrolysis: a Review. Sustainable
Energy Fuels..

[81] de
Oliveira K. L., Oliveira J. L. S., Moraes E. A. (2024). Cultivation
of Microalgae *Chlorella vulgaris*, *Monoraphidium* sp., and *Scenedesmus obliquus* in Wastewater from
the Household Appliance Industry for Bioremediation and Biofuel Production. 3 Biotechnol..

[82] Rahimi N., Eicker U. (2022). Renewable Electricity
and Hydrogen Production via Decentralized
Wastewater Treatment Systems. Energies.

[83] Heidrich E. S., Edwards S. R., Dolfing J., Cotterill S. E., Curtis T. P. (2014). Performance of a Pilot Scale Microbial
Electrolysis
Cell Fed on Domestic Wastewater at Ambient Temperatures for a 12 Month
Period. Bioresour. Technol..

[84] Holmes-Gentle I., Tembhurne S., Suter C., Haussener S. (2023). Kilowatt-Scale
Solar Hydrogen Production System Using a Concentrated Integrated Photoelectrochemical
Device. Nat. Energy.

[85] Yang Y., Bremner S., Menictas C., Kay M. (2018). Battery energy storage
system size determination in renewable energy systems: A review. Renewable Sustainable Energy Rev..

[86] Moran C., Deane P., Yousefian S., Monaghan R. F. D. (2024). The hydrogen
storage challenge: Does storage method and size affect the cost and
operational flexibility of hydrogen supply chains. Int. J. Hydrogen Energy.

[87] Eurostat . Sankey diagrams for energy balance. European Commission, 2023. https://ec.europa.eu/eurostat/statistics-explained/SEPDF/cache/50452.pdf (accessed Aug 23,2023).

[88] Enapter . AEM Nexus 1000. ENAPTER, 2023. https://handbook.enapter.com/electrolyser/aem_nexus/downloads/Enapter_Datasheet_AEM-Nexus-1000.pdf (accessed July 13, 2023).

[89] FUEL CELL ENERGY . Solid Oxide Electrolyzer (SOEC) – Spec Sheet. 2022. FuelCell Energy, Danbury, CT. Originally available at https://go.fuelcellenergy.com/hubfs/solid-oxide-electrolyzer-spec-sheet.pdf (accessed Aug 13, 2023). No longer accessible as of Oct. 2025, copy consulted from https://www.scribd.com/document/741135109/solid-oxide-electrolyzer-spec-sheet.

[90] NEL-SAS . Atmospheric Alkaline Electrolyser. 2024a. https://nelhydrogen.com/product/atmospheric-alkaline-electrolyser-a-series/ (accessed May 5, 2024).

[91] Clemens T., Hunyadi-Gall M., Lunzer A. (2024). Wind–Photovoltaic–Electrolyzer-Underground
Hydrogen Storage System for Cost-Effective Seasonal Energy Storage. Energies.

[92] Bubalo M., Bašic M., Vukadinovic D., Grgic I. (2023). Hybrid Wind-Solar Power
System with a Battery-Assisted Quasi-Z-Source Inverter: Optimal Power
Generation by Deploying Minimum Sensors. Energies.

[93] Srivastava S. (2022). Generation
of Hybrid Energy System (Solar-Wind) Supported with Battery Energy
Storage. Int. J. Res. Appl. Sci. Eng. Technol..

[94] Lave, M. ; Ellis, A. Comparison of solar and wind power generation impact on net load across a utility balancing area. In IEEE 44th Photovoltaic Specialist Conference (PVSC) Washington, DC, USA, 2017; pp 3488–3493.

[95] Clarke D. P., Al-Abdeli Y. M., Kothapalli G. (2013). The impact of renewable energy intermittency
on the operational characteristics of a stand-alone hydrogen generation
system with on-site water production. Int. J.
Hydrogen Energy..

[96] Shamim N., Subburaj A. S., Bayne S. B. (2019). Renewable
Energy Based Grid Connected
Battery Projects around the World—An Overview. J. Energy Power Eng..

[97] Kumar, K. ; Jaipal, B. The Role of Energy Storage with Renewable Electricity Generation. In Electric Grid Modernization. IntechOpen, 2022. 10.5772/intechopen.96114.

[98] Rekioua D. (2023). Energy Storage
Systems for Photovoltaic and Wind Systems: A Review. Energies.

[99] SAFT . Lithium-ion batteries in use: 5 more tips for a longer lifespan. SAFT, January 25, 2022. https://www.saft.com/energizing-iot/lithium-ion-batteries-use-5-more-tips-longer-lifespan#:~:text=Ahttps://www.saft.com/energizing-iot/lithium-ion-batteries-use-5-more-tips-longer-lifespan#:~:text=A partial charge and discharge will reduce stress and prolong, cell in your battery pack (accessed June 12, 2024).

[100] Quintino F. M., Nascimento N., Fernandes E. C. (2021). Aspects
of Hydrogen and Biomethane Introduction in Natural Gas Infrastructure
and Equipment. Hydrogen.

[101] Ingo C., Tuuf J., Björklund-Sänkiaho M. (2022). Impact of
Hydrogen on Natural Gas Compositions to Meet Engine Gas Quality Requirements. Energies.

[102] Sorgulu F., Ozturk M., Javani N., Dincer I. (2023). Experimental
investigation for combustion performance of hydrogen and natural gas
fuel blends. Int. J. Hydrogen Energy.

[103] Ferrer, S. ; Monfort, E. ; Pereira, R. ; Viduna, J. ; Montolio, J. ; Mezquita, A. ; Vedrí, J. Combustion of hydrogen-natural gas mixtures applied to the cooking of ceramic products: emissions and flame properties. Air Products and Chemicals, Inc., 2022. https://www.airproducts.co.uk/-/media/files/en/337/337-22-002-en-combustion-h2-natural-gas-mixtures-applied-cooking-eramic-products.pdf (accessed Nov 5, 2023).

[104] NEL-SAS . Frequently Asked Questions - Technical specs. 2024b. https://nelhydrogen.com/faq/ (accessed May 5, 2024).

[105] Kadier, A. ; Al-Shorgani; Najeeb, K. N. ; Jadhav, D. A. ; Sonawane, J. M. ; Mathuriya, A. S. ; Kalil, M. S. ; Hasan, H. A. ; Alabbosh, K. F. S. Microbial Electrolysis Cell (MEC): An Innovative Waste to Bioenergy and Value-Added By-product Technology. In Bioelectrosynthesis Principles and Technologies for Value-Added Products; Wiley-vch, 2020; pp 95–128 10.1002/9783527343829.ch4.

[106] SUNFIRE . PRESSURIZED ALKALINE ELECTROLYZER Sunfire-HyLink Alkaline. SUNFIRE, 2024. https://backend.sunfire.de/wp-content/uploads/2024/10/Sunfire_Fact-Sheet_AEL_EN-digital.pdf (accessed Aug 9, 2024).

[107] University of Queensland; Monash University . Green Hydrogen Production from Treated Wastewater; University of Queensland: Brisbane, Australia, 2024. https://dow.centre.uq.edu.au/files/15402/Green%20Hydrogen%20Production%20from%20Treated%20Wastewater%20April%202024.pdf. (accessed Dec 4, 2025).

[108] Merabet N.
H., Kerboua K., Hoinkis J. (2024). Hydrogen Production
from Wastewater: A Comprehensive Review of Conventional and Solar
Powered Technologies. Renewable Energy.

[109] Alqahtani H. S. (2024). Lower-Carbon Hydrogen Production
from Wastewater: A
Comprehensive Review. Sustainability.

[110] Alliat, I. The First Hythane Refueling Station In France: A Successful Demonstration. InInternational Gas Union Research Conference 2011, Proceedings and presentations, France, Paris, September 14–15, 2011. http://members.igu.org/old/IGU%20Events/igrc/igrc2011/igrc-2011-proceedings-and-presentations/poster%20paper-session%201/P1-29_Olivier%20Bordelanne.pdf.

[111] Bard, J. ; Gerhardt, N. ; Selzam, P. ; Beil, M. ; Wiemer, M. ; Buddensiek, M. limitations of hydrogen blending in the European gas grid: a study on the use, limitations and cost of hydrogen blending in the European gas grid at the transport and distribution level. Fraunhofer IEE, Berlin, 2022. https://www.iee.fraunhofer.de/content/dam/iee/energiesystemtechnik/en/documents/Studies-Reports/FINAL_FraunhoferIEE_ShortStudy_H2_Blending_EU_ECF_Jan22.pdf (accessed Aug 23, 2023).

[112] Korb B., Kawauchi S., Wachtmeister G. (2016). Influence
of hydrogen addition on the operating range, emissions and efficiency
in lean burn natural gas engines at high specific loads. Fuel.

[113] Ghimire U., Sarpong G., Gude V. G. (2021). Transitioning Wastewater
Treatment Plants toward Circular Economy and Energy Sustainability. ACS Omega..

[114] Maniscalco M. P., Longo S., Cellura M., Miccichè G., Ferraro M. (2024). Critical Review of Life Cycle Assessment
of Hydrogen
Production Pathways. Environments..

[115] NOAA, Global Monitoring Laboratory . The NOAA Annual Greenhouse Gas Index (AGGI). National Oceanic and Atmospheric Administration, Updated Summer 2024. 2023. https://gml.noaa.gov/aggi/aggi.html (accessed June 3, 2024).

[116] WHOI , Woods Hole Oceanographic Institution. Know your ocean: Ocean Acidification. 2023. https://www.whoi.edu/know-your-ocean/ocean-topics/how-the-ocean-works/ocean-chemistry/ocean-acidification/ (accessed Dec 4, 2023).

[117] GGP - Greenhouse Gas Protocol . Global Warming Potential Values. Greenhouse Gas Protocol. 2015. https://ghgprotocol.org/sites/default/files/Global-Warming-Potential-Values%28Feb162016%29_0.pdf (accessed April 18, 2023).

